# The Influence of Chemical Contaminants on the Physicochemical Properties of Unifloral and Multifloral Honey

**DOI:** 10.3390/foods10051039

**Published:** 2021-05-10

**Authors:** Laura Agripina Scripcă, Sonia Amariei

**Affiliations:** Faculty of Food Engineering, Stefan cel Mare University of Suceava, 720229 Suceava, Romania; sonia@usm.ro

**Keywords:** unifloral and multifloral honey, neonicotinoids, antibiotic residues, heavy metals

## Abstract

The aim of this study was to evaluate and compare the effect of antibiotic and pesticide residues on the physicochemical properties of unifloral and multifloral honey. The mineral elements content of honey was analyzed and correlated with antibiotic and pesticide residues, and a positive correlation was found between manganese and neonicotinoids. Potassium was found to be the most abundant mineral compound. Correlations were found between mineral content, color, and the content of antibiotic and pesticide residues of honey. In meadow honey, residues of antibiotics and pesticides were undetectable. In some of the other types of honey, the maximum residue limits regulated by European legislation were exceeded. Endosulfan residue was found in mint and rapeseed, honey with 0.42 and 5.14 ng/g, respectively. Neonicotinoids were found in 27% of the analyzed honey samples. Chloramphenicol was identified only in rapeseed honey, with concentrations ranging from 0.2 ng/g to 0.8 ng/g. Nitrofurans were found in 14%, and nitroimidazoles were found in 6% of the analyzed samples. According to EU legislation that is in force, the use of antibiotics in beekeeping is not allowed. The MRLs for neonicotinoids are 50 ng/g, and for coumaphos, the maximum limit is 100 ng/g. For the other pesticide residues, the maximum limit is 10 ng/g. The results of statistical analysis obtained using principal component analysis (PCA) showed a major difference in the levels of contamination of raspberry and meadow honey and the other types of honey.

## 1. Introduction

Honey is a sweet substance naturally produced by *Apis mellifera* bees [1]. Bees collect and transform the nectar of plants or secretions of living parts of some trees (dew), producing honey (honeydew from plant sucking insects), and storing it in honeycomb cells [2]. Honey contains more than 180 substances, and the main compounds are sugars, water, and minerals [3]. Honey also contains small amounts of phenolic compounds, vitamins, organic acids, enzymes, and amino acids [4]. Glucose and fructose are the main sugars in honey and represent more than 70% of the composition of honey [5,6]. The water content of honey ranges from 10% to 20% [7].

Mineral elements present in honey are divided into two groups, such as macroelements (calcium, potassium, magnesium, sodium) and microelements or heavy metals (iron, manganese, copper, zinc, nickel, lead, cadmium) [8,9]. The mineral content of honey ranges from 0.02% to 1.03% and represents the ash of honey [10]. Trace elements are inorganic compounds important for the vital functions of the human body [11]. Elements such as Fe, Mn, Zn, Cu, and Ni are essential for the normal functioning of the human body, but exceeding the maximum limits permitted by the legislation in force can be dangerous to human health [12]. Cadmium, lead, and nickel have carcinogenic and cytotoxic properties, which is why their presence in honey is unacceptable [13]. Mineral and heavy metal content are an indicator of the quality of honey, especially due to the toxicity of traces of heavy metals in the human body [14]. The levels of mineral elements in honey depend on botanical and geographical origin [15]. The concentration of heavy metals in honey is also influenced by soil composition [16]. Calcium, potassium, sodium, and magnesium are the main honey macroelements and account for more than 97% of the total mineral content [17].

The nutritional properties of honey are diminished if it is contaminated with toxic chemicals, such as heavy metals and pesticide residues [18]. Pesticides are toxic, and several of them are potential carcinogens [19]. Pesticides may cause changes in the endocrine system [20], the reproductive system [21,22,23], and the nervous system [24,25]. The concentration of mineral elements in honey can be determined by inductively coupled plasma mass spectrometry (ICP-MS) or by atomic absorption spectrometry (AAS) [26,27]. Honey can be contaminated with pesticides from agricultural practices and beekeeping. Plant protection products are persistent in the environment. These compounds can be transferred by nectar from the environment into honey, and it represents an indirect source of pollution. Veterinary drugs such as acaricides used by beekeepers to control Varroa destructor mite infestation of bee colonies represent the direct source of pesticide contamination of honey [28]. Residues of pesticides such as organochlorine (OCs) and organophosphorus (OPs), carbamates, and pyrethroids have been detected in honey. Residues of organophosphorus pesticides such as coumaphos and chlorpyrifos-methyl have been found in honey samples, along with organochlorine pesticide residues such as lindane, chlordane, endosulfan, aldrin, and endrin [29,30]. Human exposure to pesticides mainly occurs through diet, vegetables, fruits, but also through honey. It is estimated that humans accumulate 5 times more pesticides from food than other routes of exposure, such as air or drinking water [31]. Neonicotinoids are used for prophylactic purposes on a wide variety of crops, such as sunflower and rapeseed [32]. They are the most used insecticides for clover and account for around one-third of the worldwide insecticide market [33]. Neonicotinoid residues are found in honey because they are found in the pollen and nectar of flowering crops that are attractive to bees [34]. Imidacloprid and acetamiprid are part of the group of neonicotinoids and are the most widely used insecticides in the world [35]. The monitoring of pesticide residues in honey is therefore important to ensure honey’s quality and safety. The determination of pesticide residues in honey is performed by liquid or gas chromatography methods coupled with mass spectrometry detectors [36]. Imidacloprid and acetamiprid were approved as active substances in plant protection products by the European Union (EU), establishing maximum residue limits (MRLs) for honey [37,38,39]. Since 2013, imidacloprid has been restricted in plant protection products and treated seeds [40]. MRLs for pesticides analyzed in honey samples are established by the European Union through regulation, such as Regulation (UE) 2020/1085 [37,41,42,43,44,45].

Antibiotics are another group of contaminants that can influence the quality of honey. This group of compounds is used by beekeepers to prevent or treat bee diseases. The practice is prohibited by the European Union, and the presence of antibiotic residues in honey is also prohibited [46]. The presence of antibiotics in honey is a global problem, as they can produce residues and pose risks to human health. They can also cause allergic reactions, antibiotic resistance in humans [47], toxic effects, hepatotoxicity, and nephropathy [48,49,50]. Chloramphenicol residues can cause optic neuropathy [51]. High concentrations of nitrofurans have carcinogenic and mutagenic effects in the human body [52]. For these reasons, the use of antibiotics in food-producing animals has been banned or restricted by imposing maximum residue limits (MRLs) [53]. Commission Regulation (EU) No 37/2010 [54] has established MRLs for pharmacologically active substances in honey. According to 470/2009/CE [55] and 37/2010/CE regulations [54], the use of antibiotics in beekeeping is not allowed [56]. Residues of nitroimidazoles are substances prohibited in honey, for which the level of interest is the presence of these compounds. According to a CRL Guidance Paper [56], the recommended minimum required performance limit (MRPL) concentration of nitroimidazoles is 3 ng/mL [54].

Chloramphenicol and nitrofuran residues in honey can be determined by the ELISA technique, and nitroimidazole residues by liquid chromatography coupled with mass spectrometry [57,58,59]. MRLs for nitrofurans and their metabolites analyzed from honey samples are established by the European Union through regulation. For each metabolite of furazolidone (AOZ), furaltadone (AMOZ), nitrofurantoin (AHD), and nitrofurazone (SEM), the limit has been established at 0.5 ng/g [54,60,61]. The limits for chloramphenicol from honey samples are also established by the European Union through regulation. Residues of chloramphenicol are prohibited in honey [54,56,62]. Meadow and raspberry honey have a special chemical composition due to their protected area of origin [63]. Meadows are protected areas with several species of flowers, which are sources of polyphenols, transmitting to honey their specific aromas and colors. Mountain and sub-mountain areas (meadows), due to the absence of soil pollution, give particular properties to these types of honey in terms of both their composition and their special colors [63]. Meadow honey comes from the nectar of wildflowers in meadows from northern Romania. These wildflowers are part of a wide variety of unique flower species in the sub-mountain area.

Raspberries grow wild in relatively large areas, grouped in so-called mountain raspberry bushes. This shrub grows in deforested areas. Our study focuses attention on these types of honey and promotes efforts to maintain these protected areas from the expansion of crops treated with fertilizers and pesticides. This study also aimed to draw attention to the influence of heavy metals, pesticides, and antibiotic residues on the quality of honey.

## 2. Materials and Methods

### 2.1. Materials

The honey assortments analyzed were black locust (*Robinia pseudoacacia),* rapeseed (*Brassica napus)*, dandelion (*Taraxacum officinale)*, mint (*Mentha spicata)*, sunflower (*Helianthus annuus)*, buckwheat (*Fagopyrum esculentum)*, raspberry (*Rubus idaeus),* and meadow honey, and they were purchased from authorized local beekeepers from North Romania (commercial company—Apicola Suceava). Six samples of each assortment of honey were analyzed. The honey samples were produced in 2019. The botanical origin of the honey was guaranteed by the companies from which the samples were purchased.

The reagents used for physicochemical analysis were analytically pure. Double deionized water (18 MΩ cm resistivity) was produced by a water purification system (Thermo Fisher, Dreieich, Germany), and it was used in all solutions. Sugar standards as d (+) glucose, d (−) fructose, and d (+) sucrose were purchased from Santa Cruz Biotechnology Inc. (Dallas, TX, USA). Sigma-Aldrich (Steinheim, Germany) provided iodine solution, barbituric acid, acetic acid, and Fluka (Seelze, Germany) provided sodium carbonate. Sodium chloride solution and para-toluidine were purchased from Sigma-Aldrich (St. Louis, Missouri, USA). Acetonitrile was provided by Scharlau (Barcelona, Spain).

### 2.2. Methods

Each parameter investigated for each honey sample was determined in triplicate.

#### 2.2.1. Pesticide Residues Determination

Nine pesticide residues of three classes such as organochlorine pesticides, organophosphorus pesticides, and neonicotinoids were determined. The imidacloprid, acetamiprid, and coumaphos residues were determined by liquid chromatography (LC). The chlorpyrifos-methyl, lindane, chlordane, aldrin, endrin, and endosulfan residues were determined by gas chromatography (GC). The determination of the nine pesticide residues was performed in two stages: sample extraction and chromatographic analysis.Honey Sample Preparation for Pesticide Residues Determination by GC-MS/MS and LC-MS/MS.

The honey samples were prepared and analyzed according to the method tested by Paradis et al. [64]. The extraction of the honey samples was performed by the QuEChERS method [65]. The detailed method can be found in the Supplementary Materials, entitled Materials and Methods.

An amount of 2 μL was injected into the GC-MS/MS, and an amount of 20 μL was injected into the LC-MS/MS.

The internal standard (IS) was 1 μg/mL terbuthylazine-d5 solution.

The external standard (ES)—atrazine-d5—was at 2 mg/mL concentration.

##### GC-MS/MS Analysis

Gas chromatography analyses were performed using a gas chromatograph Agilent 7890 A with 7000 B detector with electronic impact ionization source (EI) and split/splitless injector (Agilent Technologies, Milan, Italy). A capillary column J&W HP-5ms GC column, 30 m × 0.25 mm i.d., and particle size of 0.25 µm (Agilent Technologies, Santa Clara, CA, USA) was used to separate the compounds. The detailed method can be found in the Supplementary Materials, entitled Materials and Methods. The ions were detected using a triple quadrupole mass spectrometer.

The calibration was performed with eight pesticide standard solutions.

##### LC-MS/MS Analysis

Liquid chromatography analyses were performed using a Varian Prostar system equipped with Varian 410 autosampler, pump 210, 6313-deggaser (Agilent Technologies, Oxford, UK). The chromatographic separation was carried out using the Uptisphere column C18, 150 × 2.1 mm i.d., and the particle size of 0.3 µm, preceded by a guard column Uptisphere HP Hilic 3 µm 5 × 2.1 mm Guard Cartridges (Interchim, Montluçon, France). The detection of the ions was performed using a triple quadrupole mass spectrometer. The detailed method can be found in the Supplementary Materials, entitled Materials and Methods.

The calibration was performed at eight points. Three injections were made for each calibration level.

All reagents used were pure, and they are listed in the Supplementary Materials, entitled Materials and Methods. The analytical standards for chlorpyrifos-methyl, lindane, chlordane, aldrin, endrin, and endosulfan and the Mirex standard were purchased from A ChemTek Inc. (Worcester, MA, USA). The analytical standards for acetamiprid, imidacloprid, and coumaphos were acquired from Sigma-Aldrich (Steinheim, Germany). The triphenyl phosphate, extraction salts and acetonitrile were purchased from Sigma-Aldrich (St. Louis, MO, USA).

The recovery coefficient was calculated from the formula:

(1) $$R=\frac{C_{\left(P+St\right)}\cdot C_{P}}{C_{St}}\cdot 100\left(\%\right),$$

where *C*_(*P*+*St*)_ is the sample concentration with standard added;

*C*_(*P*)_ is the sample concentration without standard; and

*C_St_* is the standard solution concentration.

The average recovery coefficient was 99.8%, and the average RSD value was 4.6%. The recoveries values of the analytical method met very well the criteria set out in guidance documents [66].

#### 2.2.2. Antibiotic Residues Determination

##### Nitroimidazoles Residues Determination by LC-MS/MS

Five nitroimidazoles were determined from forty-eight honey samples. The nitroimidazoles analyzed were metronidazole (MNZ), dimetridazole (DNZ), ronidazole (RNZ), ipronidazole (IPZ), and ipronidazole hydroxy (IPZ-OH). The identification of these five residues was performed in two steps: extraction and chromatographic analysis [67,68,69,70]. 

##### Extraction Sample

An amount of 5 g honey was weighed in a 50 mL centrifuge tube. The internal standard (MNZ-d2, DMZ-d3, RNZ-d3, IPZ-d3, IPZ-OH-d3, Witega, MNZ-13C2, 15N2, Vetranal) was added and stirred for 10 min. The mixture was purified with d-SPE using MCX (mixed cation exchange) sorbent. The sample was extracted, the supernatant was collected, and the extraction solution was evaporated to dryness in nitrogen flow (EVAP 34NGH, Organization Associates Inc., Berlin, NH, USA). The dried residue was reconstituted in 0.5 mL of 0.1% formic acid–acetonitrile (95:5, *v*/*v*) and filtered before LC-MS/MS analysis.

##### LC-MS/MS Analysis

An amount of 10 µL was injected into LC-MS/MS (Varian Prostar system, equipped with Varian 410 autosampler, pump 210, 6313-deggaser (Agilent Technologies, Oxford, UK). The separation of the compounds was performed with the column Phenomenex Synergi Polar RT 80A, 150 × 2.0 mm i.d., and the particle size was 0.4 µm. The flow rate was 300 µL/min.

The detection of the ions was performed with a triple quadrupole mass spectrometer.

All reagents used were pure and are listed in the Supplementary Materials, entitled Materials and Methods. The analytical standards for metronidazole, dimetridazole, ronidazole, ipronidazole, and ipronidazole hydroxy were purchased from Sigma-Aldrich (Steinheim, Germany). Deuterated internal standards were provided by Fluka (Vetranal, St. Louis, MI, USA) and Witega Laboratorien (Berlin, Germany).

The calibration was performed at eight points. Three injections were made for each calibration point.

The average recovery coefficient was 99.7%, and the average RSD value was 4.3%.

##### Nitrofurans Residues Determination by ELISA

Four nitrofurans were determined from forty-eight honey samples. The identified residues of nitrofurans were furazolidone (AOZ), furaltadone (AMOZ), nitrofurantoin (AHD), and nitrofurazone (SEM). Identification of the four residues was performed by enzyme-linked immunosorbent assay (ELISA) method. The analysis was performed in two steps: sample preparation and immunoenzymatic analysis [60,71,72]. 

##### Samples Extraction

The samples of honey were prepared for the determination of the four nitrofurans as follows: 1 g of homogenized sample was mixed with 1 mol/L hydrochloric acid and deionized water. After dissolving, 100 μL 10 mmol/L 2-nitrobenzaldehyde dissolved in methanol was added to the tubes for derivatization in a shaking bath at 60 °C for 2 h. An amount of 0.1 M dipotassium hydrogen phosphate and 1 M sodium hydroxide was added. Samples were then extracted. The ethyl acetate fractions were collected and dried on nitrogen flow. Residues were dissolved in 2 mL of a 1:1 (*v*/*v*) mixture of hexane and 1 mol phosphate-buffered saline (PBS, pH 7.4). The buffer phase was separated by centrifugation at 8000 rpm for 10 min. The layer aqueous phase was transferred to a vial for assay.

##### ELISA Analysis

ELISA analysis was performed according to kit directions. Enough wells were inserted into the micro-well holder for standards and samples to be determined in duplicate. Amounts of 100 μL honey sample or standard solutions were added to the wells, along with 100 μL enzyme conjugate (HRP) and 50 μL antibody solutions. The plate was incubated at room temperature (20–25 °C) for 1 h. After incubation, the liquid was poured out. Strips were washed. An amount of 100 μL 3,3′,5,5′-Tetramethylbenzidine (TMB) substrate was added to wells. The plate was incubated and stop solution was added to wells. The plate was immediately read (absorbance at 450 nm) (Molecular Devices, SpectraMax M5 microplate reader, Downingtown, PA, USA).

All reagents used were pure and are shown in the Supplementary Materials, entitled Materials and Methods. The ELISA kits were obtained from KPL Inc. (Gaithersburg, MD, USA). One kit contained microplate, nitrofuran standards at six concentrations, HRP conjugate, TMB substrate, washing solution, and stop buffer.

The calibration was performed with six standard solutions of nitrofurans: AOZ, AMOZ, AHD, and SEM.

The average recovery coefficient was 99.9%, and the average RSD value was 4.7%.

##### Chloramphenicol Residues Determination by ELISA

The chloramphenicol residues were determined by enzyme-linked immunosorbent assay (ELISA) method. The analysis was performed in two steps: sample preparation and immunoenzymatic analysis [73,74,75].

##### Extraction Sample

An amount of 2 g of each honey sample was weighed into separate centrifuge tubes. Distilled water and ethyl acetate was added to each centrifuge tube and shaken. Each tube was then centrifuged, and 1 mL of ethyl acetate supernatant was transferred into a new vial; the extract was then dried with a flow of nitrogen. The dried residue was dissolved in 0.5 mL of the buffer. A volume of 100 µL aqueous layer was used in the assay.

##### ELISA Analysis

Each standard solution-prepared blank and fortified sample was added to separate duplicate pre-coated wells in 100 µL. An amount of 100 µL of a diluted enzyme conjugate (CAP-HRP) was added to each well. The solutions were incubated for 60 min at room temperature (20–25 °C) in darkness. After incubation, the liquid was poured out. The CAP enzyme conjugate amount was visualized by adding 100 μL of TMB substrate. It was incubated for 20 min at room temperature in darkness. The absorbance was measured photometrically at 450 nm (Molecular Devices, SpectraMax M5 micro wide reader, Downingtown, PA, USA).

The calibration was performed using seven chloramphenicol standard solutions.

CAP ELISA kits were purchased from Elabscience Biotechnology (Co. Ltd., Houston, TX, USA). One kit contained microplate, chloramphenicol standards at six concentrations, HRP conjugate, TMB substrate, washing buffer, stop solution, and 2-nitrobenzaldehyde.

The recovery coefficient was calculated from Formula 1. The average recovery coefficient was 99.7%, and the average RSD value was 4.6%.

#### 2.2.3. Mineral Elements Determination

An amount of 5 g of each honey sample was weighed into the crucible and mineralized in the oven at 600 °C for 12 h until the ash became white. The ash was treated with 2 mL of concentrated nitric acid solution and 2 mL of concentrated hydrogen peroxide to destroy the organic part remaining after ashing. The sample was transferred into a 25 mL volumetric flask and rounded up to the mark with deionized water.

Mineral element analysis was performed using an inductively coupled plasma mass spectrometer (ICP-MS) from Agilent technologies 7500 (Agilent, Santa Clara, CA, USA). The ICP-MS parameters were nebulizer gas flow rate of 0.9 mL/min, RF-1500 W, carrier gas flow rate of 0.92 L/min (argon), makeup gas rate flow of 0.17 L/min, mass range 7–205 uma, integration time 0.1 s, acquisition time 22.76 s. Detector parameters: discriminator8 mV, analog HV 1770 V, and pulse HV 1070 V.

Concentration (C) of mineral elements in samples obtained is expressed in µg/g sample and is calculated using the formula [76]:

(2) $$C=c\cdot \frac{V}{m}$$

where *c* is the concentration of the element in the diluted sample (µg/g);

*V* is a total volume of diluted sample to be analyzed (mL); and

*m* is the mass of the mineralized sample (g).

The standard solutions of the elements were prepared by diluting a stock solution of 1000 mg/L of Ca, Mg, K, Na, Mn, Cu, Fe, Zn, Ni, Cd, and Pb. Stock solutions for Mg, Ca, K, Na, Mn, and Cd were purchased from Fluka (Milan, Italy), and Cu, Fe, Zn, Ni, and Pb from Merck (Darmstadt, Germany). The honey samples were treated with 65% nitric acid (Sigma-Aldrich, Darmstadt, Germany) and 30% pure hydrogen peroxide (Sigma-Aldrich, Sternheim, Germany).

Moisture content, diastase activity, and 5-hydroxymethylfurfural content (HMF) was determined according to the methods of the International Honey Commission [77,78].

##### Water Content

Water content of honey samples was determined using an Abbé Refractometer (Refractometer Re40, Mettler Toledo, Columbus, OH, USA) [79].

##### Sugars

Glucose, fructose, and sucrose were analyzed according to the Harmonized methods [78], by HPLC 10 AD VP (Shimadzu Corp., Kyoto, Japan), with a refractive detector [63].

##### Electrical Conductivity (EC)

The electrical conductivity was determined by conductometer, Mettler Toledo MPC 227 with thermostatic control [6].

##### Color Measurement

The color of the samples was determined using a chromameter, Konica Minolta CR-410 (Konica Minolta, Tokyo, Japan). L*a*b* parameters were determined by chromameter. Chromameter was calibrated with a reference white porcelain tile (L* = 97.63, a* = 0.31, b* = 4.63) before the determination. Chroma, hue angle, and yellow index were calculated using the L*a*b* value [79].

##### Viscosity

Viscosity was measured using a Brookfield viscometer, and the results are expressed in Pa·s [80].

##### Diastase Activity Determination

The analysis of diastase activity was performed by determining the activity of amylase. The diastase index is defined as the number of milliliters of 1% starch solution that was processed in dextrin for one hour at 45 °C at an optimum pH of amylase containing 1 g honey. The diastase activity was determined by the Schade procedure [26,81]. The absorbance was measured at a wavelength of 660 nm using a Perkin Elmer UV-VIS LAMBDA EZ201.

##### Hydroxymethylfurfural Concentration

Hydroxymethylfurfural (HMF) was determined by the Winkler method. HMF concentration was determined using a Perkin Elmer UV-VIS LAMBDA EZ201 equipped with cuvettes with a layer of 1 cm thickness and at a wavelength of 550 nm. The color intensity of the red complex formed by HMF with barbituric acid in the presence of para-toluidine is proportional to the concentration of HMF [82,83].

#### 2.2.4. Statistical Analysis

The results were analyzed by principal component analysis (PCA) and descriptive analyses with XLSTAT software (2020, trial version, Long Island, NY, USA). PCA evaluated the correlations between the honey type, physicochemical parameters, minerals, and antibiotic and pesticide residues and extracted the main components. Pearson correlations between the variables were calculated using Pearson’s coefficient (a *p*-value < 0.05 was considered statistically significant). The significance level alpha was 0.05.

## 3. Results and Discussion

Physicochemical parameters of eight types of honey (black locust, rapeseed, dandelion, mint, sunflower, buckwheat, raspberry, and meadow) were investigated. Table 1 and Table 2 and Tables S1–S3 present the quality parameters. The physicochemical parameters determined were water content, sugars, electrical conductivity, diastase activity, HMF, viscosity, and color. Mineral elements analyzed were sodium (Na), potassium (K), magnesium (Mg), calcium (Ca), manganese (Mn), copper (Cu), iron (Fe), zinc (Zn), nickel (Ni), lead (Pb), and cadmium (Cd). Pesticide residues investigated were imidacloprid, acetamiprid, lindane, aldrin, endrin, chlordane, endosulfan, coumaphos, and chlorpyrifos-methyl. Antibiotic residues investigated were nitroimidazoles residues (dimetridazole, DMZ; ronidazole, RNZ; metronidazole, MNZ; ipronidazole, IPZ: and ipronidazole-hydroxy, IPZ-OH), nitrofuran residues (furazolidone, AOZ; furaltadone, AMOZ; nitrofurantoin, AHD; nitrofurazone, SEM), and chloramphenicol residues (CAP).

### 3.1. Pesticide Residues

**Figure 1:**
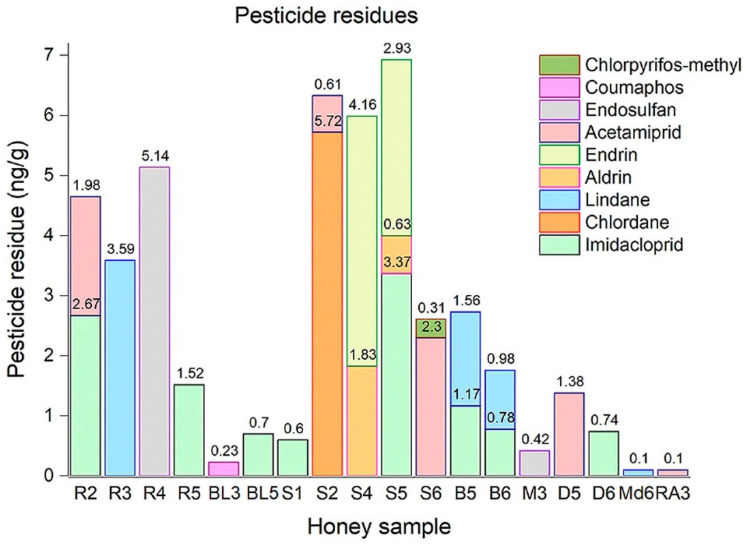
The pesticide residues concentration in different honey samples (R, rapeseed honey; BL, black locust; S, sunflower; B, buckwheat; M, mint; D, dandelion; Md, meadow; RA, raspberry).

The results of pesticide residues obtained for analyzed honey samples are shown in Table S3 and in Figure 1.

The pesticide residues analyzed were imidacloprid, acetamiprid, coumaphos, chlorpyrifos-methyl, lindane, chlordane, aldrin, endrin, and endosulfan. The MRLs for neonicotinoids are 50 ng/g, and for coumaphos the maximum limit is 100 ng/g. The other pesticide residue levels should be below 10 ng/g. Neonicotinoids were most commonly detected in the honey samples analyzed. Different concentrations of pesticide residues were found in 48 honey samples analyzed. The main source of the detected pesticides was agricultural application. The lowest pesticide content was found in meadow and raspberry samples, which prove that these samples were safe for consumers, came from wild areas, and could perhaps mean a lack of pesticide treatment. This fact distinguished them from other types of honey analyzed. A small amount of acetamiprid (G6 with 0.10 ng/g) was found in a single sample of meadow honey. In raspberry honey, a small concentration of lindane was found (RA3 with 0.10 ng/g). Residues of imidacloprid and acetamiprid were found in most of the positive samples; their highest concentrations were found in sunflower honey. No pesticide residues were detected in more than 60% of the analyzed samples. Residues of imidacloprid were detected in approximately 16% of the analyzed sample honey. Acetamiprid residues were detected in approximately 10% of the analyzed samples. In one sample (S5), three pesticide residues were detected. Two pesticide residues were detected in 26% of the positive samples. In 68% of samples confirmed with pesticide presence, one residue of pesticides was detected. Residues of imidacloprid (R2, R5), acetamiprid (R2), lindane (R3), and endosulfan (R4) were found in rapeseed honey. The endosulfan residue had the highest concentration (5.147 ng/g). Chlorpyrifos-methyl, chlordane, aldrin, endrin, imidacloprid, and acetamiprid were found in sunflower honey samples. Neonicotinoid residues were found in two samples of dandelion honey. All results were according to the MRLs provided by European legislation and did not exceed the maximum limit.

González-Miret et al. [84] presented correlations between the Mn content and the color parameters for dark honey but also for light honey samples. The authors reported higher manganese concentrations and lower brightness values in honeydew and chestnut honey than in the other lighter varieties of honey. In our case, the honey samples with higher brightness also contained Mn and showed traces of neonicotinoids. Achanta et al. [85] reported that mean L* values of yogurts fortified with manganese, magnesium, chromium, and molybdenum were higher than the control samples. Couto et al. [86] reported higher values of the L* parameter (40.0 ± 7.7) and lower concentrations of Mn (0.1 g/100 g) in centrifuged pineapple juice than in concentrated juice (L* = 25.1 ± 2.7, Mn = 0.5 g/100 g). These results are confirmed by statistical analysis of the Pearson correlation performed. In conclusion, manganese content could influence color parameters, or the presence of neonicotinoid residues in honey could be a factor influencing both manganese content and color parameters.

Valdovinos-Flores et al. [87] analyzed 172 samples of honey collected from three areas of Mexico and found residues of imidacloprid in 3 samples. In another study, Bommuraj et al. [88] analyzed honey samples from Israel and reported the presence of imidacloprid with an average concentration of 7 ng/g and acetamiprid with an average concentration 2.3 ng/g. The prevalence of these residues was 65.6% for imidacloprid and 15% for acetamiprid. Nadaf et al. [89] reported the presence of pesticide residues in 28 of 30 analyzed samples. Aldrin, endosulfan, chlorpyrifos-methyl, and lindane residues were similar to our study, but the concentrations found were much higher than those found by us. Aldrin was found in 53% of the analyzed samples, and it ranged from 93 ng/g to 283 ng/g. Chlorpyrifos methyl was found in ten samples and ranged between 32 ng/g and 83 ng/g. Contrary to the results obtained in this study, Pravcová et al. [90] reported that meadow honey was found to have the highest concentration of acetamiprid (17 mg/kg). Acacia and forest honey had the lowest concentration (0.24 and 0.22 mg/kg). Deng et al. [91] analyzed eighteen kinds of commercial honey samples purchased from Metro supermarket, Nanchang, China. Chlorpyrifos methyl was found in all rapeseed and clover honey samples and in one wildflower honey sample, and all concentrations of this residue were approximately 10 times higher than reported in this study. Rafique et al. [92] reported the detection of nine pesticide residues in samples of honey from 26 Pakistani apiaries. These residues were found in almost 27% of the samples analyzed. The average concentrations of lindane and chlorpyrifos-methyl were much higher than those reported in this study (26.9 and 17.8 mg/kg). Coumaphos was not detected in any honey sample analyzed. Song et al. [93] identified several pesticide residues, including acetamiprid and imidacloprid, and reported, contrary to our study, much higher concentrations. Acetamiprid incidence was 6, the concentration varied between 28 and 68 ng/g. The incidence of imidacloprid was 5 and concentration ranged from 28 to 72 ng/g. Bargańska et al. [94] collected and analyzed fifteen Polish honey samples. Pesticide residues were identified in the multifloral and rapeseed honey samples. Chlorpyrifos-methyl is an insecticide of the acaricide group and was found in 93.3% of honey samples. Fifty samples of honey collected from different areas of Argentina were analyzed by Medici et al. [95], and the presence of endosulfan was reported in ten samples. Ninety-eight honey samples collected from Italy were analyzed by Panseri et al. [96]. Coumaphos was detected in one sample, with a concentration of 1.64 ng/g.

### 3.2. Antibiotic Residues

**Figure 2:**
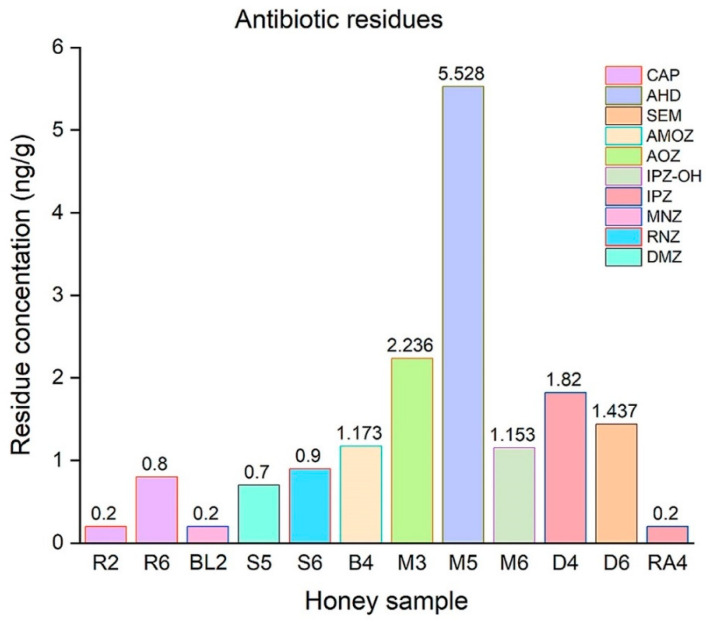
The antibiotic residues concentration in different honey samples (CAP, chloramphenicol; AHD, nitrofurantoin; SEM, nitrofurazone; AMOZ, furaltadone; AOZ, furazolidone; IPZ-OH, ipronidazole-hydroxy; IPZ, ipronidazole; MNZ, metronidazole; RNZ, ronidazole; DMZ, dimetridazole).

The results of antibiotic residues obtained for analyzed honey samples are shown in Table S2 and Figure 2. The antibiotic residues analyzed were five nitroimidazoles (DMZ, RNZ, MNZ, IPZ, and IPZ-OH), four nitrofurans (AOZ, AMOZ, SEM, and AHD), and chloramphenicol (CAP). Ipronidazole and chloramphenicol was identified in two samples each. Different concentrations of antibiotic residues were found in 48 honey samples analyzed. The meadow samples were free from antibiotic residues. The lowest antibiotic concentration (0.2 ng/g each) was found in black locust and raspberry honey. Antibiotic residue incidence was 25%. No antibiotic residues were detected in 75% of the analyzed samples. Three antibiotic residues (IPZ-OH, AOZ, and AHD) were detected in mint honey samples. Chloramphenicol (R2, R6) and ipronidazole (D4, RA3) were found in two samples each. AHD was found with the highest concentration (5.528 ng/g) in mint honey sample M5. Antibiotic residues are banned in honey, which means that 25% of the samples analyzed were contaminated with antibiotic residues. Within the EU, antibiotic residues are not allowed, but there is no determined maximum residue level, and traces can be found in honey samples worldwide [97].

Petcu et al. [98] reported nitrofurans, nitroimidazoles, and chloramphenicol residues below the quantification limit for black locust honey samples. Rimkus et al. [75] reported similar results, with CAP concentrations ranging between 0.2 ng/g and 1.1 ng/g for sunflower honey collected from Ukraine and Bulgaria. In the buckwheat honey sample, CAP concentration was 0.1 ng/g. Chiesa et al. [99] reported that no CAP, AOZ, or AMOZ residues were found in Italian honey. In another study, Morariu et al. [100] reported a high incidence of CAP residues in Romanian honey, with a concentration between 0.07–2.34 ng/g. Jia et al. [101] identified similar results as our study for nitrofurans in multifloral honey collected from China. The concentration of AHD was 2.14 ± 0.09 ng/g, for AMOZ it was 1.83 ± 0.03 ng/g, for AOZ it was 2.50 ± 0.03 ng/g, and for SEM it was 2.16 ± 0.04 ng/g. Lei et al. [69] analyzed acacia honey and jujube honey and reported residues of metronidazole with a concentration between 5.87 ng/g and 66.95 ng/g in two samples of each. Jin et al. [102] reported similar results for DMZ (0.46 ng/g), MNZ (0.50 ng/g), and RNZ (2.42 ng/g). Of the 3855 honey samples analyzed, antibiotics such as chloramphenicol (0.1–169 ng/g) and nitrofurans (0.3–24.7 ng/g) were identified in 1.7% of the samples [103]. The results of an antibiotic residues investigation in 135 honey samples collected from Iran showed that the chloramphenicol had the lowest level of concentration (0.1 ng/g) [104].

### 3.3. Mineral Elements

**Figure 3:**
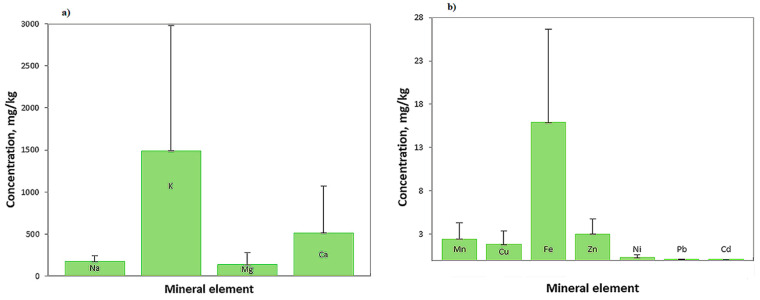
The mean concentration of macroelements (**a**) and of microelements (**b**) for eight types of honey (Na, sodium; K, potassium; Mg, magnesium; Ca, calcium; Mn, manganese; Cu, copper; Fe, iron; Zn, zinc; Ni, nickel; Pb, lead; Cd, cadmium).

The results obtained for analyzed honey samples are shown in Table S1 and Figure 3. The mineral elements analyzed were sodium (Na), potassium (K), magnesium (Mg), calcium (Ca), manganese (Mn), copper (Cu), iron (Fe), zinc (Zn), nickel (Ni), lead (Pb), and cadmium (Cd). Minerals are important because their concentration in honey depends on nutrients and soil substances that can reach honey through the nectar harvested from bee plants [105,106]. Simultaneously, the composition of minerals in honey depends on the climatic conditions and geographic area [107]. The mineral content of honey is also important because it is an important parameter in the authentication and classification of honey according to the botanical origin of the nectar [108].

Potassium was the most abundant mineral element in analyzed samples. The highest concentration (5528 mg/kg) was found in dandelion honey, and the lowest concentration was found in buckwheat honey. Analyzed samples of raspberry honey and meadow honey were found to have undetectable Cd and Pb values. Low values of Ni were found in two samples of meadow honey (Md1 with 0.02 mg/kg and Md3 with 0.01 mg/kg) and two samples of raspberry honey (RA1 and RA6 with 0.01 mg/kg each). These results prove that the samples came from an ecological, clean, unpolluted area that was suitable for production. The lowest concentrations of Ca characterized all black locust honey samples, and Cd was not detected in four samples. Sunflower honey samples were characterized by the lowest Mn content (0.75 mg/kg) and by the highest Fe level (37.01 mg/kg). The highest concentrations of Na (339.17 mg/kg), Mn (7.65 mg/kg), Zn (8.01 mg/kg), and Cd (0.08 mg/kg) and the lowest concentrations of Cu (0.23 mg/kg) and Fe (3.25 mg/kg) were found in buckwheat honey samples. The highest concentrations of Mg (427.92 mg/kg), Ca (1603.45 mg/kg), Ni (1.02 mg/kg), Pb (0.33 mg/kg), and Cd (0.08 mg/kg) characterized mint honey samples. The lowest level of Na (72.02 mg/kg), Mg (6.45 mg/kg), Zn (0.59 mg/kg), and by the highest level of Cu (9.67 mg/kg) characterized dandelion honey samples.

Sajtos et al. [109] analyzed 187 honey samples collected from different pollen and nectar-producing parts of Hungary and reported that in sunflower analyzed honey was found the highest content of Ca (217 mg/kg) and Zn (5.5 mg/kg) and the lowest concentration of Mn (0.7 mg/kg). In black locust honey was found the lowest K level (327.9 mg/kg), and in rapeseed honey was found the lowest mg content (7.2 mg/kg). In another study, Jovetić et al. [110] reported that potassium was the most abundant metal in all 206 samples of five floral types of honey investigated (200 to 1543 mg/kg), and the next most abundant element was calcium (27.3–87.0 mg/kg). The calcium content in sunflower samples was 2 times higher than that in rapeseed samples and 4 times higher than in acacia honey samples. Czipa et al. [111] reported higher average concentrations of Cd for sunflower samples (1.15 μg/kg) and for black locust samples (0.736 μg/kg) due to the intensive use of fertilizers. The smallest amount of Pb, Cd, and Ni was found in honey samples harvested from mountain meadows, away from the road and factories, which is attributed to the honey’s uncontaminated environment [112]. Similar results were obtained in our study for meadow and raspberry honey samples. Aghamirlou et al. [113] determined the concentrations of some metals in Iranian honey and reported that the most abundant metal in honey samples was Zn, with 1481.64 μg/kg. Uršulin-Trstenjak et al. [114] reported similar results for Zn, Ni, and Pb concentrations determined for 200 black locust honey samples collected from five regions of Croatia. Dżugan et al. [115] analyzed Polish honey samples from different botanical origin, such as dandelion, rapeseed, and buckwheat. Similar to our results, potassium was the dominant macroelement; the lowest concentration was found in rapeseed samples, with 310.59 mg/kg. Nowak et al. [116] reported high concentrations of Mn and Cu in buckwheat honey and low concentrations of Mn in rapeseed honey due to the capacity of buckwheat to absorb some of these elements from the soil and accumulate them.

### 3.4. Water Content

The water content of honey is a quality criterion. Honey with lower water content is stable for a longer period [117]. According to European Directive 2001/110/EC, the water content should not exceed 20% in honey [1]. Honey easily absorbs humidity from the environment [17]. High moisture content can also be determined by late harvesting of honey or early harvesting of unripe honey [118]. The high-water content in honey causes fermentation and changes its taste and stability [119,120]. The glucose/water content ratio (G/W) is an indicator of honey crystallization. Honey crystallizes faster when G/W ratio is greater than 2.1 [121].

Water content values in the analyzed honey samples were normal and did not exceed 20%. All results of water content complied with the legislation established by the Codex Alimentarius [122]. The lowest water content was found in buckwheat (B) and sunflower (S) samples—B2 and S1, with 15.9% each. The highest content was found in black locust (BL) honey BL6, with 19.5%. The results obtained for the water content from the analyzed samples indicate good stability over time, and the risk of fermentation processes is lower in buckwheat and sunflower honey than in black locust samples. The results show that rapeseed honey has the highest crystallization capacity (G/W = 2.18–2.47), and black locust honey has the lowest crystallization rate (G/W = 1.39–1.62). Buckwheat honey has a relatively small crystallization capacity, with G/W ratio values close to black locust honey. The other types of honey tend to crystallize more quickly, as their G/W ratios were around 2.0.

Comparable results to the results of our study were reported by Abdulkhaliq and Swaileh [123] and Boussaid et al. [124]. Abdulkhaliq reported water content values for 33 samples of multifloral honey between 14.5% and 19.0% [123]. Boussaid et al. [124] reported values ranging from 17.27% to 19.80% for six honey samples from different botanical origins harvested in Tunisia. Water content in Anatolian sample honey was reported by Küçük et al. [125]; the values ranged from 19.0% to 19.7%. Marghitas et al. [126] reported moisture content values for Romanian honey between 16.6% and 20.0%. Baloš et al. [127] reported similar results for acacia, meadow, and sunflower honey water content (black locust ranging from 13.8% to 19.0%; meadow ranging from 14.0% to 19.0%, and sunflower ranging from 15.6% to 18.6%). Özenirler [128] reported water content as 14.5% in dandelion honey. Raspberry honey water content ranged from 17.5% to 20.4%, and for dandelion honey, water content values were between 17.0% and 24.9% [129]. Kędzierska-Matysek et al. [130] analyzed and reported results similar to ours, finding that rapeseed honey had a water content between 17.4% and 19.4%. Saeed and Jayashankar [131] found G/W values for black locust honey equal to 1.70 ± 0.34. Baloš et al. [132] reported results similar to those in our study for average values of G/W ratio for meadow honey (1.98 ± 0.17) and black locust honey (1.75 ± 0.22).

### 3.5. Sugars

Three sugars were investigated from forty-eight samples of eight types of honey. The results are shown in Table 1. Glucose and fructose were the main compounds in the honey samples analyzed but were found in different ratios from one assortment to the next. Sugar content of honey depends on the botanical origin of honey [133]. The glucose to fructose ratio (G/F) is used to evaluate honey crystallization because glucose is less soluble in water than fructose [134]. A higher G/F ratio than 1.0 causes honey to crystallize faster [135]. Honeys with a G/F ratio lower than 1.0 tends to remain liquid for a longer period [136]. The G/F ratio depends on the species of honeybee [137]. Sucrose content is one of the parameters used to determine the authenticity of honey. The high sucrose content in honey is due to early harvest of the product, as sucrose from nectar has not yet been broken down into glucose and fructose by enzymes [138]. Adulteration with different syrups is linked to a high sucrose content of honey. According to the Codex Alimentarius [122], the sucrose content of honey should not exceed 5 g/100 g.

The lowest level of glucose concentration was found in black locust sample BL2, with 25.13 g/100 g honey. The highest concentration of glucose was 42.27 g/100 g in rapeseed sample R6. Fructose content ranged from 27.39 g/100 g honey in rapeseed honey sample R2 to 47.48 g/100 g honey in black locust sample BL3. Buckwheat honey samples analyzed had high fructose content ranging from 41.99 g/100 g to 42.47 g/100 g. The other types of honey analyzed had a concentration of fructose and glucose around 34–35 g/100 g. The G/F ratio was smaller than 1.0 for the analyzed black locust, buckwheat, dandelion, and raspberry honeys. These honeys have a lesser tendency to crystallize. The lower ratio was for black locust honey and was equal to 0.54 for A5. Some samples of sunflower honey (S1) had a G/F < 1.0, and some samples (S2–S6) had a G/F > 1.0. A greater G/F ratio characterized rapeseed and meadow samples (1.32–1.51 for rapeseed honey and 1.12–1.19 for meadow honey).

All samples analyzed had a sucrose content of less than 5%, which could indicate that the honey samples were properly matured before harvesting and were not adulterated by the addition of syrup. The highest level of sucrose was in S4, with 3.82 g/100 g, and the lowest concentration of sucrose was found in A1, with 0.17 g/100 g. The results obtained in this study confirmed earlier studies [139,140]. Yayinie et al. [141] found sucrose values between 2.96 ± 0.30 and 4.73 ± 0.09 g/100 g for the honey samples analyzed.

Deng et al. [142] found an average glucose content of 34.8 ± 0.35 g/100 g and an average fructose content of 36.4 ± 0.14 g/100 g in buckwheat honey harvested from Chaoyang, Liaoning Province, China. In another study, Tedesco et al. [143] reported higher glucose content in dandelion honey than in other honey samples. Sunflower honey samples usually contain a large amount of glucose, similar to our results [144].

Sakač et al. [145], reported similar results from those presented in this study, with the sugar content of three types of honey: black locust, meadow, and sunflower. The glucose content was between 27.8 and 33.1 g/100 g in black locust honey, 28.4 and 33.8 in meadow honey, and 29.4 and 34.1 g/100 g in sunflower honey. The fructose content was between 31.7 and 39.4 g/100 g in black locust honey, 31.8 and 36.2 in meadow honey, and 31.8 and 36.2 g/100 g in sunflower honey.

Salonen et al. [146] reported that the average value of the G/F ratio was 1.22 ± 0.02 for raspberry honey. Escuredo et al. [147] reported a G/F ratio of 1.02 for sunflower honey, 1.13 for rapeseed honey, and 1.17 for lime honey. The results obtained in our study are in accordance with the results reported by these scientists.

### 3.6. Electrical Conductivity

Electrical conductivity (EC) indicates the ability of honey to conduct an electric current. This parameter depends on the mineral content of honey [148]. It is the most useful quality parameter for the classification of monofloral honey [149]. According to European legislation, honey electrical conductivity should not exceed 0.8 mS/cm—except for *Tilia* sp., *Erica* sp., *Eucalyptus* sp., Calluna, and Manuka, which have higher electrical conductivity than 0.8 mS/cm. Honeydew honey always has electrical conductivity over 0.8 mS/cm [150]. It is considered a good criterion for identifying the botanical origin of honey, but it is also used in routine control of honey quality [151].

All EC values for the analyzed samples followed the current European legislation. The lowest level of electrical conductivity was found in rapeseed honey (R2 with 0.13 mS/cm), and the highest level was 0.56 mS/cm in raspberry sample RA5.

Similar results to those obtained by us were found by Ceylan et al. [152] for sunflower honey (EC = 0.27 mS/cm). Conti et al. [153] reported that the average electrical conductivity values of multifloral honey from five regions of Italy were equal to 0.63 ± 0.37 mS/cm.

### 3.7. Color

The color of honey is one of the most important indicators for consumers. Lightly colored honey is preferred over dark honey. Floral origin and the nectar source can influence honey color [154]. The age, storage conditions, processing and harvesting of honey in old or new combs found in the hive, and time in the formation of the comb in the beehive cause modification of honey color [155]. Dark and amber honey has a higher content of certain minerals (Na, K, Ca, Mg, Fe, Cu, Zn, Al, Ni, Cd, and Mn) compared with lightly colored honey [156]. Metal transition influences the color of honey by forming complexes with some organic compounds. Additionally, thermal processing can influence the color of honey due to the formation of Maillard’s reaction products [157]. Darker honey has the highest antioxidant capacity [158].

The results obtained for analyzed honey samples are shown in Table 2. The color parameters analyzed were L*, a*, b* (L*, luminosity; positive values of a* (red) and negative values of a* (green); positive values of b* (yellow) and negative values of b* (blue)), the chroma (C- saturation), the hue angle (H), and the yellow index (YI).

The L* parameter represents the lightness of honey, and the parameter ranged between 17.61 in rapeseed honey to 52.39 in black locust honey. The a* parameter represents the green compound (negative a* values), which was present in all samples of black locust, rapeseed, and mint honey. The highest value of the green compound was found in mint honey (−3.50). The red parameter (positive a* values) was found in sunflower, buckwheat, dandelion, meadow, and raspberry honey. The highest value of the red parameter was found for meadow honey (7.23). The yellow parameter (positive b* values) was found in all samples and in all types of honey analyzed. These values ranged between 3.45 in mint honey and 17.37 in buckwheat honey. Chroma (C) ranged from rapeseed sample R2 with 4.56 to buckwheat sample B5 with 17.37. Hue angle (H) ranged from rapeseed sample R4 with 1.54 to buckwheat samples B3 and B4 with 1.55 each. The lowest yellow index value was found in mint honey, with 17.52, and the highest value was in raspberry honey, with 78.01.

The color of the honey samples was characterized by shades of red, yellow, and green, with the samples of sunflower, buckwheat, dandelion, meadow, and raspberry honey being located in the first quadrant of the CIE color space a* b* because the coordinates a* and b* had positive values. The negative values of the parameter a* of black locust, rapeseed, and mint honey samples place them in the second quadrant, and they are characterized by shades of yellow and green.

Popov-Raljić et al. [159] analyzed black locust and meadow honey samples from Serbia and reported results for color. L* parameter range from 24.48 to 31.64 for meadow honey and from 25.10 to 32.59 for black locust honey. The a* parameter varied between −6.71 and 3.98 for meadow honey and between −1.19 and −8.03 for black locust honey. The b* parameter ranged from 8.18 to 12.67 for meadow honey and from 6.84 to 20.00 for black locust honey. In another study, Kaczmarek et al. [160] reported similar results for the color parameter of black locust, buckwheat, rapeseed, and multifloral honey. For black locust honey, the color parameter reported was L* = 51 ± 5.6, a* = −3.41 ± 0.44, b* = 18.6 ± 4.59, H = −0.19 ± 0.05, and C = 18.9 ± 4.49. For buckwheat honey, the color parameter reported was L* = 33 ± 8.7, a* = 2.25 ± 3.84, b* = 8.39 ± 3.48, H = 0.22 ± 0.35, and C = 9.29 ± 3.88. For rapeseed honey, the color parameter reported was L* = 43 ± 0.5, a* = −3.03 ± 0.36, b* = 16.32 ± 0.25, H = −0.18 ± 0.02, and C= 16.6 ± 0.29. For multifloral honey, the color parameter reported was L* = 42 ± 1.9, a* = −1.14 ± 1.05, b* = 23.7 ± 8.99, H = 0.05 ± 0.04, and C = 22.74 ± 9. Bayram et al. [161] analyzed 60 honey samples collected from the 12 regions of the Bayburt province of Turkey and obtained similar results to those obtained in our study. L* parameter varied from 20.06 to 29.73, a* parameter varied from 0.85 to 3.25, and b* parameter varied from −3.67 to −7.80. Belay et al. [162] reported that the color parameter values for Ethiopian black locust honey: L* parameter was 51.09 ± 0.08, a* parameter was −0.45 ± 0.01, and b* parameter was 4.60 ± 0.10—results that were in accordance with our result.

### 3.8. Viscosity

Viscosity is an important property of honey in the evaluation of fluidity and crystallization and can influence other physicochemical or organoleptic properties of honey [163]. The rheological properties of honey are important in processing, handling, and storage, while high viscosity honey causes problems in handling and processing [164]. The viscosity (V) of honey depends on factors such as temperature, water content, chemical constitution, quantity, and size of crystals present in it [165]. Moreover, sugars, non-sugar content, and colloidals produce differences in viscosity [166,167].

The highest value of viscosity (11.59 Pa·s) was found in rapeseed honey, and the lowest level (3.59 Pa·s) of this parameter was found in black locust honey. The values obtained for the viscosity were correlated with glucose content. High levels of glucose cause high viscosity in rapeseed honey due to low glucose solubility. In the black locust, mint, and raspberry samples, the viscosity was determined by higher fructose content than in other varieties of honey.

The viscosity values found in our study were higher than the values reported by Leme et al. [168] (0.41–1.61). This author investigated honey samples harvested from Roraima, in the north of Brazil, and Paraná, in the south of Brazil. Lullah-Deh [80] reported viscosity results ranging from 23.2 to 34.7 Pa·s for honey samples harvested from the bee farmers and purchased in the market. The viscosity of the honey from the beekeepers had higher values than that of the market honey. Al-Habsi et al. [169] analyzed four types of honey (Brazilian, Australian, Chinese, and Romanian black locust honey) and reported that the higher viscosity value was Romanian honey, with 36.50 Pa·s, and the lower value was for Brazilian honey, with 3.13 Pa·s. The Chinese honey viscosity was 8.03 Pa·s, and the viscosity of Australian honey was 34.40 Pa·s.

### 3.9. Diastase Activity

The enzyme diastase, or α-amylase, is an indicator of honey freshness, and it is thermosensitive [170]. Diastasic activity is also an indication of the storage time of honey [171]. The diastatic activity of honey is correlated with the source of nectar and with the geographical origin of honey [172,173,174]. Results are expressed in Göthe units per gram of honey [122].

The highest value of diastase activity of the analyzed samples was found in raspberry honey sample RA1 with 24.28° Göthe. The lowest values were in rapeseed honey sample R5 with 11.36° Göthe. All samples had diastase activity values higher than 8.0, indicating that the quality of the analyzed honey samples was high.

Similar results to the results obtained in our study were reported by Da Silva et al. [175]. The diastase activity values reported ranged from 11.14° to 22.69° Göthe for Brazilian honey. Diastase activity of honey ranged from 6.02° to 21.99° Göthe [176]. Kamal et al. [177] reported values of diastase activity ranging from 12.63° to 16.33° Göthe for five honey samples harvested from Bangladesh. Khalafi et al. [178] reported values of diastase activity between 5.8° and 21.3° Göthe for ten types of honey. Isopescu et al. [179] analyzed Romanian honey samples and reported diastase activity results. For black locust honey, diastase activity ranged from 6.5° to 29.4° Göthe, for sunflower honey from 23.8° to 29.4° Göthe, and for rapeseed honey from 8.3° to 23.8° Göthe.

### 3.10. HMF Concentration

HMF content is an indicator of the freshness of honey [180]. It is an important parameter with which to evaluate honey quality. HMF is found naturally in small quantities in honey, but its concentration can increase because of heating treatments applied to honey [181]. Hydroxymethylfurfural has a cytotoxic, genotoxic, and organotoxic effect in humans [182]. Therefore, the European Commission has established a maximum HMF concentration of 40 mg/kg (80 mg/kg in tropical honey) [1,122].

The HMF content values of the analyzed samples were below the maximum limits allowed by the legislation, which proves the freshness of all analyzed samples but also proves the lack of heating treatment. The lowest level of HMF was found in mint honey (M1 with 0.11 mg/kg), and the highest level was 4.47 mg/kg in rapeseed sample R5.

Rajs et al. [183] reported values of HMF ranging from 3.0 to 15.3 mg/kg for 21 rapeseed honey samples from different areas of Croatia. HMF content was reported by Dżugan et al. [184]; the values ranged from 0.77 to 14.40 mg/kg. The authors reported results for HMF content much higher than the results we obtained.

### 3.11. Statistical Results

Pearson correlation (Supplementary Materials Table S4) was performed for each type of analyzed honey. Table S4a (rapeseed honey) showed that there are only positive correlations between sucrose and HMF content (0.910), between sucrose and diastase activity (0.806), between HMF and diastase activity (0.924), and between Mn and Na (0.881). Table S4b (black locust honey) presents a positive correlation of cadmium with sodium (0.923); of copper with G/F ratio (0.829) and magnesium (0.821); of water content with HMF (0.963) and electrical conductivity (0.806); and a negative correlation between cadmium and G/W content ratio (−0.847), a* parameter (−0.823), and calcium (−0.867); between manganese and calcium (−0.835) and L* parameter (0.996); between sodium and G/W content ratio (−0.903), ID (−0.875), and a* parameter (−0.908); and between HMF and diastase activity (−0.977). In Table S4c (sunflower honey), positive correlations were observed between L* parameter copper (0.820) and between lead and conductivity. A negative correlation was found between a* parameter and calcium. Only positive correlations existed in Table S4d (buckwheat honey) between zinc and L* (0.819) and a* (0.825) parameters; between cadmium and glucose (0.829), fructose (0.943), sucrose (0.881), and yellow index (0.896); between nickel and diastase activity (0.822); and between electrical conductivity and chroma (0.899), b* parameter (0.899). Table S4e with the correlations obtained for the analyzed samples of mint honey showed only positive correlations between magnesium and nickel (0.993); between HMF and iron (0.830); and between glucose and lead (0.834) and cadmium (0.802). Table S4f (dandelion honey) showed positive correlations between iron and sucrose (0.804) and HMF (0.800); between manganese and copper (0.801); and between fructose and diastase activity (0.940). Table S4g with the correlations obtained for the analyzed samples of meadow honey showed positive correlations between magnesium and a* parameter (0.852) and hue angle (0.834); between sucrose and potassium (0.801); between iron and sodium (0.952); and between L* parameter and glucose (0.824) and fructose (0.909). Table S4h (raspberry honey) presents positive correlations of a* parameter with nickel (0.804), of copper with sodium (0.863), and of sucrose with diastase activity (0.932).

**Figure 4:**
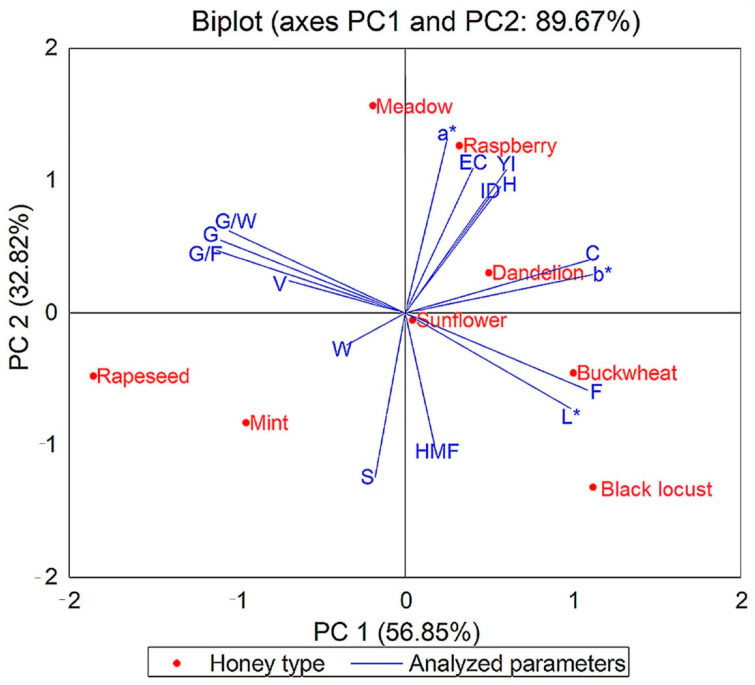
Principal component analysis (PCA) of dataset consisting of physicochemical analyzed parameters of each honey sample (G, glucose; F, fructose; W, water content; EC, electrical conductivity; YI, yellow index; H, hue angle; ID, diastase activity; C, chroma; HMF, hydroxymethylfurfural; S, sucrose; V, viscosity; L*, a*, b*, color parameters).

**Figure 5:**
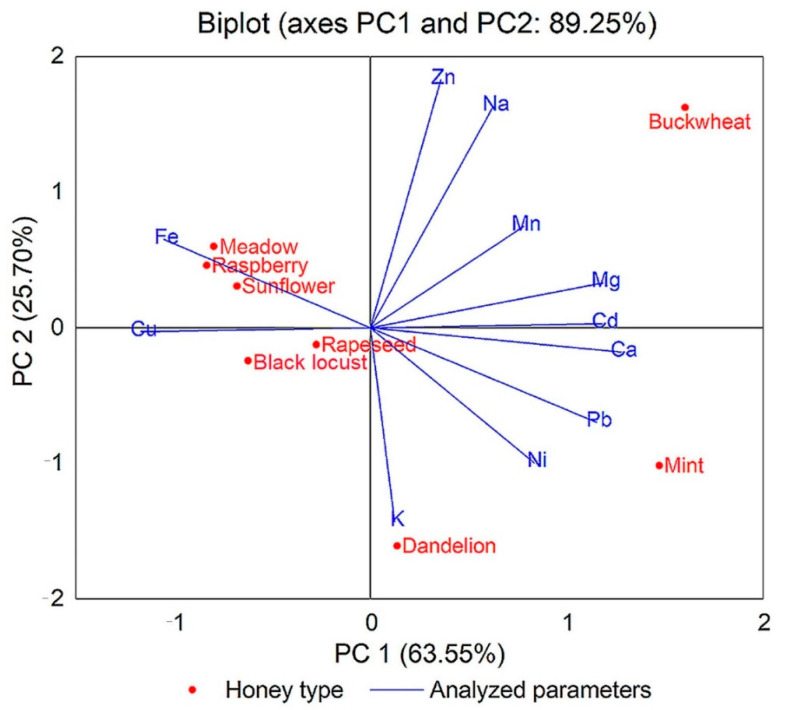
Principal component analysis (PCA) of dataset consisting of mineral elements analyzed of each honey sample (Na, sodium; K, potassium; Mg, magnesium; Ca, calcium; Mn, manganese; Cu, copper; Fe, iron; Zn, zinc; Ni, nickel; Pb, lead; Cd, cadmium).

**Figure 6:**
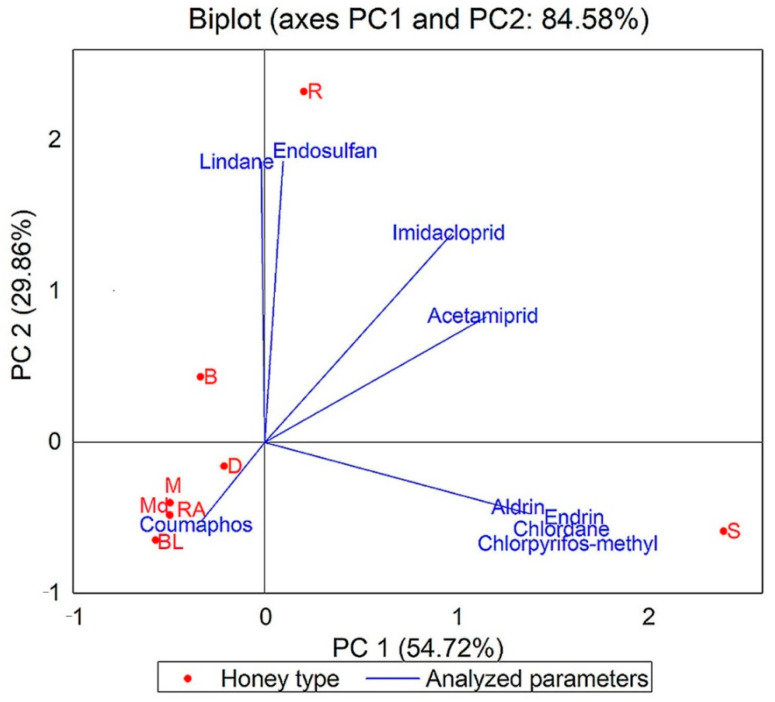
Principal component analysis (PCA) of dataset consisting of pesticide residues analyzed of each honey sample (BL, black locust honey samples; B, buckwheat; R, rapeseed; S, sunflower; D, dandelion; M, mint; Md, meadow; RA, raspberry).

**Figure 7:**
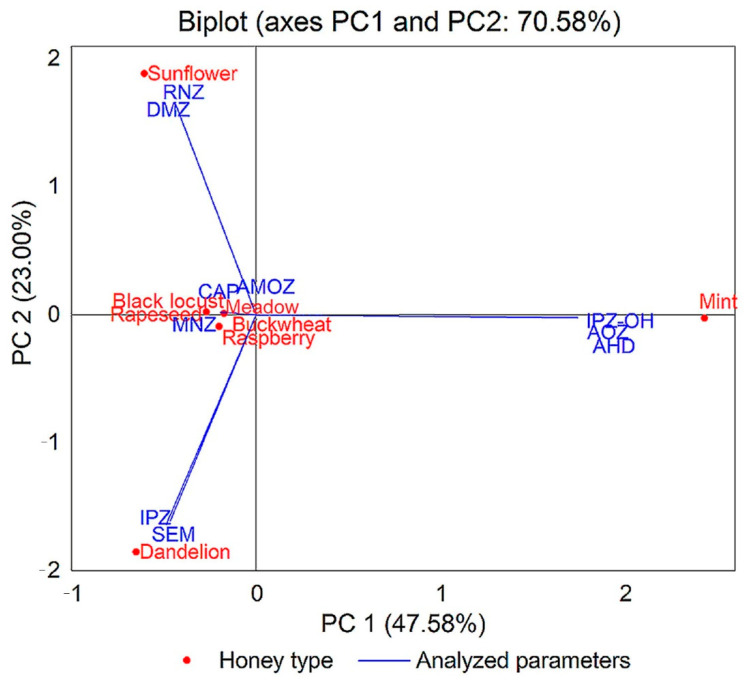
Principal component analysis (PCA) of dataset consisting of antibiotic residues analyzed of each honey sample (CAP, chloramphenicol; AHD, nitrofurantoin; SEM, nitrofurazone; AMOZ, furaltadone; AOZ, furazolidone; IPZ-OH, ipronidazole-hydroxy; IPZ, ipronidazole; MNZ, metronidazole; RNZ, ronidazole; DMZ, dimetridazole).

To emphasize the difference between the eight types of honey and the parameters analyzed, we performed an analysis of the main components (PCA). The results are presented in the biplot in Figure 4, Figure 5, Figure 6 and Figure 7. The PCA method limited all data in two main components, the active variables being water content (W), glucose content (G), fructose content (F), G/F ratio, G/W ratio, diastase activity (ID), hydroxymethylfurfural concentration (HMF), and some color parameters, mineral elements, and pesticide and antibiotic residues.

The PCA method narrowed the data into two main components covering 89.67% (for the physicochemical results obtained), 89.25% (for the results of mineral composition of eight types of honey), 84.58% (for the results of pesticide residues), and 70.58% (for the results of antibiotic residues) of the variance.

From the PCA illustrated in Figure 4, it can be observed that sunflower honey, buckwheat honey, mint, meadow, raspberry, and dandelion honey have similarities in results, with higher values of electrical conductivity, color parameters, diastase activity, glucose, G/F ratio, G/W content ratio than black locust and rapeseed honey.

Figure 5 shows the distribution of mineral elements, which indicates that the iron and copper had higher values and characterize raspberry, sunflower, rapeseed, black locust, and meadow honey. On the opposite side of the graph, dandelion and mint honey had lower values of the parameters listed above. These figures showed a significant difference in these eight types of honey, a difference that consists of high content of potassium in dandelion honey and of lead, nickel, and calcium in mint honey.

From the PCA illustrated in Figure 6, it can be observed that the aldrin, endrin, chlordane, and chlorpyrifos-methyl residues characterize sunflower honey and that lindane and endosulfan residues characterize rapeseed honey.

From the PCA illustrated in Figure 7, it can be observed that dimetridazole and ronidazole residues are characteristic of sunflower honey; ipronidazole-hydroxy, furazolidone, and nitrofurantoin residues are characteristic of mint honey; and ipronidazole and nitrofurazone residues are characteristic of dandelion honey.

## 4. Conclusions

The meadow and raspberry honey samples analyzed have superior quality to the other varieties of honey analyzed. Meadow and raspberry are special honeys because of their physicochemical properties, and they also have special properties in terms of their lower degree of contamination with pesticide residues, antibiotics, or heavy metals. The meadow honey samples, through the results obtained, did not contain heavy metals or antibiotic residues compared with the other varieties of honey, except for raspberry honey. Samples contaminated with pesticides, antibiotics, or heavy metals have, in most cases, higher quantities of HMF and color changes compared with the uncontaminated samples of the same assortment. Therefore, the presence of contaminants in some samples influences the values of their physicochemical parameters. The manganese concentration and the brightness of the honey samples analyzed are related to the presence of neonicotinoids in these samples.

All samples analyzed had a sucrose content of less than of 5%, the maximum limit regulated by European legislation, which could indicate that the honey was not adulterated with sugar syrup and was properly matured before harvesting.

## Figures and Tables

**Table 1:** Physicochemical parameters of different types of honey from Romania.

Honey Variety		Water Content, %	Glucose, g/100 g	Fructose, g/100 g	Sucrose, g/100 g	G/F	G/W	Conductivity, mS/cm	HMF, mg/kg	Diastase Activity, (°)	Viscosity, Pa·s
Rapeseed	Mean ± SD	17.63 ± 0.36	41.31 ± 0.38	29.36 ± 0.53	1.80 ± 0.44	1.41 ± 0.03	2.34 ± 0.05	0.16 ± 0.01	1.98 ± 0.74	15.48 ± 1.13	10.51 ± 0.07
	Min	16.7	39.79	27.39	0.56	1.32	2.18	0.13	0.24	11.36	9.79
	Max	19	42.27	31.07	3.15	1.51	2.47	0.19	4.47	18.3	11.59
Black locust	Mean ± SD	17.96 ± 0.55	27.03 ± 0.61	43.60 ± 0.95	1.96 ± 0.49	0.61 ± 0.04	1.50 ± 0.03	0.24 ± 0.02	1.99 ± 0.75	20.39 ± 1.40	4.30 ± 0.02
	Min	16.3	25.13	40.18	0.76	0.54	1.39	0.17	0.19	16.49	3.58
	Max	19.5	29.16	47.48	3.04	0.69	1.56	0.35	4.14	24.18	5.09
Sunflower	Mean ± SD	17.34 ± 0.30	34.76 ± 0.05	34.51 ± 0.17	2.32 ± 0.59	0.82 ± 0.18	2.00 ± 0.15	0.35 ± 0.08	2.10 ± 0.12	17.71 ± 0.45	7.45 ± 0.05
	Min	16.5	34.7	33.82	0.78	0.1	1.93	0.28	0.72	13.86	7.02
	Max	17.9	34.98	34.71	3.82	1.03	2.1	0.47	4.11	21.06	7.87
Buckwheat	Mean ± SD	16.18 ± 0.08	28.58 ± 0.09	42.28 ± 0.07	2.29 ± 0.08	0.67 ± 0.03	1.76 ± 0.09	0.39 ± 0.06	2.27 ± 0.05	17.16 ± 0.37	8.63 ± 0.06
	Min	15.9	28.35	42.02	0.63	0.66	1.73	0.3	1.38	14.99	8.05
	Max	16.4	28.87	42.47	3.63	0.68	1.81	0.49	3.17	19.24	9.21
Mint	Mean ± SD	17.24 ± 0.41	35.22 ± 0.06	33.35 ± 0.10	2.71 ± 0.07	1.054 ± 0.02	2.04 ± 0.05	0.39 ± 0.01	1.83 ± 0.11	16.85 ± 0.99	6.25 ± 0.09
	Min	16.2	35.05	33.04	2.16	1.04	1.93	0.36	0.78	15.08	5.23
	Max	18.2	35.37	33.55	3.51	1.07	2.17	0.42	2.88	20.04	6.93
Dandelion	Mean ± SD	16.76 ± 0.15	33.32 ± 0.25	36.78 ± 0.45	0.67 ± 0.12	0.90 ± 0.18	1.98 ± 0.09	0.34 ± 0.02	2.51 ± 0.02	17.20 ± 0.24	7.37 ± 0.07
	Min	16.4	33.22	36.69	0.19	0.9	1.94	0.27	0.96	16.83	6.89
	Max	17.1	33.45	36.83	0.99	0.91	2.04	0.43	3.94	18.14	7.89
Meadow	Mean ± SD	17.10 ± 0.33	37.10 ± 0.12	32.15 ± 0.23	0.25 ± 0.10	1.15 ± 0.09	2.17 ± 0.06	0.51 ± 0.05	0.25 ± 0.01	22.68 ± 0.32	7.45 ± 0.04
	Min	16.3	36.42	32.04	0.12	1.12	2.03	0.48	0.14	21.98	6.03
	Max	17.9	37.52	32.33	0.46	1.17	2.29	0.55	0.43	23.47	8.13
Raspberry	Mean ± SD	17.36 ± 0.19	34.28 ± 0.31	35.41 ± 0.26	0.68 ± 0.12	0.97 ± 0.02	1.97 ± 0.32	0.51 ± 0.09	0.71 ± 0.05	21.40 ± 0.30	6.69 ± 0.11
	Min	16.8	33.64	34.92	0.32	0.94	1.88	0.46	0.18	18.25	6.06
	Max	17.9	35.61	35.77	1.02	1.00	2.06	0.56	1.26	24.28	7.04

Values are expressed as means ± SD, *n* = 6; SD, standard deviations; Min, minimum value; Max, maximum value; G/F, glucose to fructose ratio; G/W, glucose to water content ratio.

**Table 2:** Color parameters of different types of honey from Romania.

Honey Variety		L*	a*	b*	Chroma	Hue angle (°)	Yellow Index
Rapeseed	Mean ± SD	24.38 ± 1.58	−0.45 ± 0.03	6.32 ± 0.57	6.34 ± 0.12	−1.50 ± 0.08	38.97 ± 0.16
	Min	17.61	−1.25	4.56	4.56	−1.54	25.91
	Max	29.02	−0.16	8.17	8.26	−1.41	66.27
Black locust	Mean ± SD	48.52 ± 0.65	−1.15 ± 0.07	14.90 ± 0.18	14.95 ± 0.10	−1.49 ± 0.02	43.97 ± 0.27
	Min	45.96	−1.69	14.18	13.3	−1.52	39.37
	Max	52.39	−0.83	16.34	16.36	−1.48	50.48
Sunflower	Mean ± SD	37.48 ± 0.58	2.22 ± 0.29	12.32 ± 0.28	12.62 ± 0.23	1.38 ± 0.02	47.84 ± 0.19
	Min	36.22	1.25	11.45	11.51	1.34	41.83
	Max	39.1	2.98	13.15	13.48	1.46	51.83
Buckwheat	Mean ± SD	42.85 ± 0.65	0.35 ± 0.08	16.10 ± 0.07	15.90 ± 0.15	1.54 ± 0.05	53.03 ± 0.26
	Min	40.7	0.21	14.17	14.17	1.54	49.73
	Max	44.85	0.51	17.37	17.37	1.55	55.33
Mint	Mean ± SD	28.43 ± 0.16	−2.67 ± 0.12	5.87 ± 0.22	6.29 ± 0.06	−1.09 ± 0.05	28.73 ± 0.21
	Min	26.97	−3.50	3.45	4.91	−1.32	17.52
	Max	29.93	−1.89	7.62	7.85	−0.77	36.37
Dandelion	Mean ± SD	33.62 ± 0.97	1.19 ± 0.25	16.07 ± 0.15	16.28 ± 0.18	1.49 ± 0.06	68.49 ± 0.32
	Min	33.06	0.87	15.25	15.31	1.47	65.89
	Max	34.49	1.56	17.13	17.15	1.52	70.95
Meadow	Mean ± SD	27.28 ± 0.48	5.00 ± 0.29	11.85 ± 0.30	13.06 ± 0.26	1.20 ± 0.10	60.42 ± 0.19
	Min	21.56	1.07	9.67	12.07	0.92	52.04
	Max	32.14	7.23	14.09	14.61	1.49	64.07
Raspberry	Mean ± SD	27.88 ± 0.13	5.11 ± 0.12	13.73 ± 0.27	14.66 ± 0.12	1.21 ± 0.04	70.48 ± 0.35
	Min	26.15	4.22	12.73	13.41	1.17	64.53
	Max	29.28	5.81	14.62	15.73	1.25	78.01

Values are expressed as means ± SD, *n* = 6; SD: standard deviations; Min, minimum value; Max, maximum value; L*, lightness of honey; a*, from red (+) to green (−); b*, from yellow (+) to blue (-); Chroma, saturation.

## Data Availability

Data available on request.

## Supplementary Materials

The following are available online at https://www.mdpi.com/article/10.3390/foods10051039/s1, Supplementary Materials: Materials and methods; Table S1: Mineral elements of analyzed honey samples, Table S2: Antibiotic residues of analyzed honey samples, Table S3: Pesticide residues of analyzed honey samples. Table S4: Pearson correlation for physicochemical and minerals of different type of honey.
