# Supplementary material for: The Influence of Chemical Contaminants on the Physicochemical Properties of Unifloral and Multifloral Honey
Source: Foods. 2021 May 10;10(5):1039. doi: 10.3390/foods10051039 (PMC8150977; doi:10.3390/foods10051039)
Supplement: Supplementary file 1 [file foods-10-01039-s001.zip › foods-1185281-supplementary.pdf]

Table S1. Supplement material – Mineral elements of analyzed honey samples

Honey Variety		Multielement, mg/kg										
		**Na**	**K**	**Mg**	**Ca**	**Mn**	**Cu**	**Fe**	**Zn**	**Ni**	**Pb**	**Cd**
Rapeseed	R1	152.60±0.10	812.72±0.23	61.19±0.61	161.97±0.11	1.03±0.01	2.33±0.38	23.89±0.15	3.21±0.27	0.78±0.01	0.08±0.03	0.02±0.02
	R2	162.13±0.15	817.28±0.32	65.98±0.03	175.89±0.14	1.43±0.04	2.09±0.06	23.05±0.13	3.20±0.05	0.49±0.08	0.04±0.02	0.04±0.02
	R3	155.28±0.14	810.82±0.23	64.29±0.07	156.09±0.10	1.26±0.06	1.98±0.05	24.37±0.21	3.01±0.02	0.63±0.06	0.02±0.01	0.01±0.01
	R4	150.32±0.12	815.12±0.18	61.09±0.12	159.67±0.12	1.09±0.05	2.22±0.06	22.46±0.07	2.67±0.03	0.57±0.02	0.05±0.02	0.03±0.01
	R5	148.29±0.21	819.23±0.02	71.49±0.04	164.27±0.10	0.97±0.10	2.76±0.01	22.98±0.06	3.54±0.01	0.48±0.03	0.01±0.02	0.02±0.02
	R6	151.93±0.06	809.02±0.18	70.28±0.14	266.98±0.15	1.01±0.04	2.13±0.04	23.48±0.05	3.24±0.05	0.79±0.02	0.02±0.01	0.01±0.02
Black locust	BL1	103.50±0.26	468.75±0.72	44.39±0.53	51.71±0.03	1.14±0.25	1.63±0.59	12.80±0.47	1.76±0.08	0.03±0.01	0.01±0.01	0.00±0.01
	BL2	108.14±0.18	463.98±0.26	44.98±0.07	55.12±0.06	0.95±0.03	1.67±0.05	12.02±0.03	1.98±0.04	0.03±0.02	0.01±0.01	0.00±0.01
	BL3	105.25±0.03	454.38±0.06	44.09±0.14	52.09±0.10	0.98±0.05	1.45±0.02	11.98±0.09	1.56±0.05	0.01±0.01	0.01±0.02	0.00±0.01
	BL4	123.58±0.15	473.91±0.14	44.89±0.02	49.03±0.07	1.19±0.02	1.78±0.05	11.45±0.02	1.87±0.01	0.02±0.02	0.00±0.00	0.01±0.02
	BL5	118.64±0.07	471.12±0.10	43.11±0.15	45.98±0.01	1.57±0.03	1.38±0.02	12.76±0.07	1.39±0.02	0.02±0.10	0.01±0.01	0.01±0.02
	BL6	111.12±0.14	466.41±0.15	46.92±0.01	52.13±0.12	1.32±0.04	1.77±0.04	13.02±0.01	1.23±0.05	0.03±0.02	0.01±0.01	0.00±0.01
Sunflower	S1	148.34±0.02	638.58±0.18	52.17±0.07	102.27±0.15	0.86±0.02	1.56±0.05	28.24±0.07	2.62±0.02	0.14±0.01	0.02±0.01	0.00±0.01
	S2	155.22±0.14	630.28±0.12	55.29±0.12	108.05±0.14	0.76±0.06	1.67±0.10	28.14±0.02	2.76±0.03	0.09±0.02	0.01±0.01	0.01±0.02
	S3	150.48±0.10	622.15±0.04	51.98±0.14	97.13±0.03	0.75±0.05	1.98±0.06	32.09±0.10	3.18±0.02	0.01±0.01	0.01±0.02	0.00±0.01
	S4	145.89±0.12	637.84±0.36	49.99±0.06	99.41±0.07	0.87±0.08	2.19±0.03	37.01±0.09	2.33±0.01	0.10±0.01	0.01±0.01	0.01±0.01
	S5	145.36±0.01	652.46±0.03	54.36±0.13	145.02±0.10	0.82±0.01	2.09±0.05	30.56±0.18	3.98±0.03	0.17±0.04	0.03±0.02	0.01±0.01
	S6	147.72±0.12	654.19±0.15	50.12±0.07	129.56±0.15	0.93±0.02	1.63±0.06	24.31±0.25	5.03±0.07	0.15±0.03	0.01±0.01	0.03±0.02
Buckwheat	B1	321.12±0.10	398.54±0.30	329.09±0.18	1286.43±0.65	6.27±0.06	0.25±0.03	3.94±0.07	7.22±0.12	0.22±0.02	0.13±0.03	0.01±0.02
	B2	325.29±0.18	390.06±0.12	333.07±0.10	1256.90±0.12	6.45±0.10	0.43±0.05	4.98±0.04	8.01±0.06	0.17±0.01	0.04±0.02	0.01±0.01
	B3	298.38±0.17	379.28±0.04	374.10±0.14	1198.04±0.37	6.97±0.06	0.29±0.01	4.28±0.06	5.08±0.09	0.06±0.03	0.02±0.02	0.05±0.03
	B4	339.17±0.12	401.16±0.18	396.46±0.02	1265.34±0.18	6.75±0.06	0.23±0.04	4.08±0.08	6.08±0.10	0.32±0.05	0.01±0.01	0.08±0.03
	B5	320.08±0.14	383.58±0.01	317.59±0.06	1298.65±0.42	5.12±0.05	0.56±0.02	3.67±0.04	4.67±0.06	0.23±0.01	0.08±0.01	0.07±0.02
	B6	299.76±0.18	396.57±0.12	297.42±0.12	1209.74±0.28	7.65±0.01	0.28±0.01	3.25±0.07	7.09±0.07	0.43±0.02	0.18±0.03	0.08±0.03
Mint	M1	163.67±0.06	1873.69±0.35	408.82±0.15	1590.17±0.12	0.97±0.07	0.32±0.02	3.64±0.01	1.17±0.02	0.87±0.07	0.08±0.02	0.02±0.03
	M2	185.35±0.22	1801.03±0.18	400.38±0.34	1529.75±0.48	1.45±0.04	0.65±0.05	3.56±0.05	1.87±0.02	0.80±0.03	0.01±0.01	0.00±0.01
	M3	166.13±0.15	1639.59±0.03	356.98±0.10	1593.01±0.25	2.15±0.06	0.37±0.04	3.92±0.07	2.67±0.03	0.45±0.01	0.00±0.00	0.00±0.01
	M4	171.17±0.04	1708.73±0.37	428.63±0.18	1538.57±0.36	1.78±0.01	0.28±0.06	4.19±0.05	1.87±0.01	0.98±0.03	0.32±0.03	0.05±0.03
	M5	164.92±0.18	1812.48±0.10	419.06±0.14	1409.29±0.04	0.87±0.05	0.47±0.03	4.87±0.04	1.64±0.06	0.96±0.12	0.29±0.06	0.03±0.02
	M6	161.72±0.15	1789.36±0.45	427.92±0.15	1603.45±0.46	0.99±0.01	0.45±0.04	4.67±0.06	1.79±0.07	1.02±0.03	0.33±0.03	0.08±0.01

Table S1. Cont.

Honey Variety		Multielement, mg/kg										
		**Na**	**K**	**Mg**	**Ca**	**Mn**	**Cu**	**Fe**	**Zn**	**Ni**	**Pb**	**Cd**
Dandelion	D1	74.17±0.12	5110.34±0.33	9.87±0.06	611.23±0.14	3.61±0.06	0.36±0.06	4.21±0.02	0.65±0.04	0.35±0.02	0.09±0.02	0.03±0.01
	D2	83.15±0.10	4912.08±0.26	15.08±0.12	600.54±0.10	3.24±0.07	0.56±0.03	4.56±0.01	0.60±0.01	0.39±0.03	0.05±0.03	0.03±0.03
	D3	77.28±0.14	4538.95±0.28	6.45±0.03	587.93±0.22	3.87±0.02	0.55±0.05	4.29±0.07	0.64±0.06	0.33±0.01	0.03±0.01	0.02±0.02
	D4	76.59±0.05	5528.09±0.25	20.18±0.12	607.19±0.14	3.68±0.06	0.49±0.04	3.29±0.10	0.59±0.01	0.41±0.09	0.08±0.05	0.05±0.03
	D5	72.02±0.04	5361.72±0.37	17.42±0.14	599.02±0.27	3.98±0.04	9.67±0.05	4.01±0.06	0.71±0.03	0.42±0.01	0.09±0.03	0.02±0.01
	D6	79.47±0.14	5004.65±0.65	13.67±0.16	562.48±0.10	4.19±0.03	0.44±0.06	3.82±0.04	0.66±0.05	0.39±0.11	0.01±0.03	0.03±0.03
Meadow	Md1	214.86±0.18	1648.28±0.36	69.79±0.09	113.56±0.45	2.01±0.72	3.08±0.13	24.16±0.06	3.01±0.67	0.02±0.03	0.00±0.01	0.00±0.01
	Md2	214.09±0.12	1600.06±0.41	72.17±0.07	121.38±0.12	2.18±0.07	3.54±0.09	23.98±0.02	3.17±0.06	0.00±0.00	0.00±0.01	0.00±0.00
	Md3	245.76±0.04	1828.54±0.22	66.38±0.14	117.47±0.10	2.09±0.06	3.19±0.07	34.17±0.06	3.74±0.07	0.01±0.01	0.00±0.01	0.00±0.00
	Md4	213.07±0.10	1505.56±0.15	69.39±0.15	120.10±0.10	1.78±0.12	2.89±0.04	25.49±0.18	3.28±0.11	0.00±0.00	0.00±0.00	0.00±0.00
	Md5	205.69±0.19	1643.27±0.10	72.89±0.12	108.13±0.17	1.96±0.02	2.76±0.15	22.39±0.10	4.19±0.04	0.00±0.01	0.00±0.00	0.00±0.01
	Md6	208.37±0.14	1543.57±0.36	72.14±0.18	118.26±0.13	2.19±0.05	3.02±0.07	24.58±0.02	4.02±0.05	0.00±0.01	0.00±0.00	0.00±0.01
Raspberry	RA1	164.86±0.09	792.28±0.35	52.45±0.21	89.22±0.14	1.98±0.66	2.87±0.69	25.49±0.04	3.08±0.31	0.01±0.03	0.00±0.00	0.00±0.01
	RA2	166.11±0.15	790.47±0.17	55.19±0.14	90.58±0.12	2.11±0.06	2.88±0.05	24.17±0.17	3.04±0.07	0.00±0.01	0.00±0.00	0.00±0.00
	RA3	170.37±0.10	815.49±0.27	50.28±0.04	86.13±0.05	2.56±0.02	2.34±0.09	26.98±0.04	3.48±0.16	0.00±0.00	0.00±0.01	0.00±0.01
	RA4	167.52±0.14	802.75±0.10	49.09±0.12	89.28±0.12	1.78±0.07	2.56±0.04	20.94±0.10	3.23±0.06	0.00±0.01	0.00±0.00	0.00±0.01
	RA5	164.23±0.05	783.68±0.28	48.98±0.07	96.52±0.23	1.95±0.06	3.09±0.06	25.84±0.28	2.98±0.07	0.00±0.00	0.00±0.00	0.00±0.00
	RA6	165.18±0.12	760.39±0.19	60.35±0.10	92.15±0.14	2.08±0.04	2.74±0.05	25.30±0.07	3.65±0.13	0.01±0.03	0.00±0.01	0.00±0.00

Every value is a mean of three determinations (n = 3) ± standard deviations;

Table S2. Supplement material – Antibiotic residues of analyzed honey samples

Honey Variety		Antibiotic residues, ng/g									
		Nitroimidazoles					Nitrofurans				Chloramphenicol
		DMZ	RNZ	MNZ	IPZ	IPZ-OH	AOZ	AMOZ	SEM	AHD	CAP
Rapeseed	R1	n.d.	n.d.	n.d.	n.d.	n.d.	n.d.	n.d.	n.d.	n.d.	n.d.
	R2	n.d.	n.d.	n.d.	n.d.	n.d.	n.d.	n.d.	n.d.	n.d.	0.200±0.001
	R3	n.d.	n.d.	n.d.	n.d.	n.d.	n.d.	n.d.	n.d.	n.d.	n.d.
	R4	n.d.	n.d.	n.d.	n.d.	n.d.	n.d.	n.d.	n.d.	n.d.	n.d.
	R5	n.d.	n.d.	n.d.	n.d.	n.d.	n.d.	n.d.	n.d.	n.d.	n.d.
	R6	n.d.	n.d.	n.d.	n.d.	n.d.	n.d.	n.d.	n.d.	n.d.	0.800±0.003
Black locust	BL1	n.d.	n.d.	n.d.	n.d.	n.d.	n.d.	n.d.	n.d.	n.d.	n.d.
	BL2	n.d.	n.d.	0.200±0.001	n.d.	n.d.	n.d.	n.d.	n.d.	n.d.	n.d.
	BL3	n.d.	n.d.	n.d.	n.d.	n.d.	n.d.	n.d.	n.d.	n.d.	n.d.
	BL4	n.d.	n.d.	n.d.	n.d.	n.d.	n.d.	n.d.	n.d.	n.d.	n.d.
	BL5	n.d.	n.d.	n.d.	n.d.	n.d.	n.d.	n.d.	n.d.	n.d.	n.d.
	BL6	n.d.	n.d.	n.d.	n.d.	n.d.	n.d.	n.d.	n.d.	n.d.	n.d.
Sunflower	S1	n.d.	n.d.	n.d.	n.d.	n.d.	n.d.	n.d.	n.d.	n.d.	n.d.
	S2	n.d.	n.d.	n.d.	n.d.	n.d.	n.d.	n.d.	n.d.	n.d.	n.d.
	S3	n.d.	n.d.	n.d.	n.d.	n.d.	n.d.	n.d.	n.d.	n.d.	n.d.
	S4	n.d.	n.d.	n.d.	n.d.	n.d.	n.d.	n.d.	n.d.	n.d.	n.d.
	S5	0.700±0.002	n.d.	n.d.	n.d.	n.d.	n.d.	n.d.	n.d.	n.d.	n.d.
	S6	n.d.	0.900±0.001	n.d.	n.d.	n.d.	n.d.	n.d.	n.d.	n.d.	n.d.
Buckwheat	B1	n.d.	n.d.	n.d.	n.d.	n.d.	n.d.	n.d.	n.d.	n.d.	n.d.
	B2	n.d.	n.d.	n.d.	n.d.	n.d.	n.d.	n.d.	n.d.	n.d.	n.d.
	B3	n.d.	n.d.	n.d.	n.d.	n.d.	n.d.	n.d.	n.d.	n.d.	n.d.
	B4	n.d.	n.d.	n.d.	n.d.	n.d.	n.d.	1.173±0.027	n.d.	n.d.	n.d.
	B5	n.d.	n.d.	n.d.	n.d.	n.d.	n.d.	n.d.	n.d.	n.d.	n.d.
	B6	n.d.	n.d.	n.d.	n.d.	n.d.	n.d.	n.d.	n.d.	n.d.	n.d.
Mint	M1	n.d.	n.d.	n.d.	n.d.	n.d.	n.d.	n.d.	n.d.	n.d.	n.d.
	M2	n.d.	n.d.	n.d.	n.d.	n.d.	n.d.	n.d.	n.d.	n.d.	n.d.
	M3	n.d.	n.d.	n.d.	n.d.	n.d.	2.236±0.051	n.d.	n.d.	n.d.	n.d.
	M4	n.d.	n.d.	n.d.	n.d.	n.d.	n.d.	n.d.	n.d.	n.d.	n.d.
	M5	n.d.	n.d.	n.d.	n.d.	n.d.	n.d.	n.d.	n.d.	5.528±0.043	n.d.
	M6	n.d.	n.d.	n.d.	n.d.	1.153±0.022	n.d.	n.d.	n.d.	n.d.	n.d.

Table S2. Cont.

Honey Variety		Antibiotic residues, ng/g									
		Nitroimidazoles					Nitrofurans				Chloramphenicol
		DMZ	RNZ	MNZ	IPZ	IPZ-OH	AOZ	AMOZ	SEM	AHD	CAP
Dandelion	D1	n.d.	n.d.	n.d.	n.d.	n.d.	n.d.	n.d.	n.d.	n.d.	n.d.
	D2	n.d.	n.d.	n.d.	n.d.	n.d.	n.d.	n.d.	n.d.	n.d.	n.d.
	D3	n.d.	n.d.	n.d.	n.d.	n.d.	n.d.	n.d.	n.d.	n.d.	n.d.
	D4	n.d.	n.d.	n.d.	1.820±0.002	n.d.	n.d.	n.d.	n.d.	n.d.	n.d.
	D5	n.d.	n.d.	n.d.	n.d.	n.d.	n.d.	n.d.	n.d.	n.d.	n.d.
	D6	n.d.	n.d.	n.d.	n.d.	n.d.	n.d.	n.d.	1.437±0.005	n.d.	n.d.
Meadow	Md1	n.d.	n.d.	n.d.	n.d.	n.d.	n.d.	n.d.	n.d.	n.d.	n.d.
	Md2	n.d.	n.d.	n.d.	n.d.	n.d.	n.d.	n.d.	n.d.	n.d.	n.d.
	Md3	n.d.	n.d.	n.d.	n.d.	n.d.	n.d.	n.d.	n.d.	n.d.	n.d.
	Md4	n.d.	n.d.	n.d.	n.d.	n.d.	n.d.	n.d.	n.d.	n.d.	n.d.
	Md5	n.d.	n.d.	n.d.	n.d.	n.d.	n.d.	n.d.	n.d.	n.d.	n.d.
	Md6	n.d.	n.d.	n.d.	n.d.	n.d.	n.d.	n.d.	n.d.	n.d.	n.d.
Raspberry	RA1	n.d.	n.d.	n.d.	n.d.	n.d.	n.d.	n.d.	n.d.	n.d.	n.d.
	RA2	n.d.	n.d.	n.d.	n.d.	n.d.	n.d.	n.d.	n.d.	n.d.	n.d.
	RA3	n.d.	n.d.	n.d.	0.200±0.001	n.d.	n.d.	n.d.	n.d.	n.d.	n.d.
	RA4	n.d.	n.d.	n.d.	n.d.	n.d.	n.d.	n.d.	n.d.	n.d.	n.d.
	RA5	n.d.	n.d.	n.d.	n.d.	n.d.	n.d.	n.d.	n.d.	n.d.	n.d.
	RA6	n.d.	n.d.	n.d.	n.d.	n.d.	n.d.	n.d.	n.d.	n.d.	n.d.

Every value is a mean of three determinations (n = 3) ± standard deviations; DMZ- dimetridazole; RNZ- ronidazole; MNZ- metronidazole; IPZ- ipronidazole; IPZ-OH- ipornidazole-hydroxy; AOZ- furazolidone; AMOZ- furaltadone;SEM- Nitrofurazon; AHD- Nitrofurantoin; CAP- chloramphenicol; n.d.-undetectable.

Table S3. Supplement material – Pesticide residues of analyzed honey samples

Honey Variety		Pesticide residues, ng/g								
		**Imidacloprid**	**Acetamiprid**	**Lindane**	**Aldrin**	**Endrin**	**Chlordane**	**Endosulfan**	**Coumaphos**	**Chlorpyrifos-methyl**
Rapeseed	R1	n.d.	n.d.	n.d.	n.d.	n.d.	n.d.	n.d.	n.d.	n.d.
	R2	2.672±0.002	1.980±0.001	n.d.	n.d.	n.d.	n.d.	n.d.	n.d.	n.d.
	R3	n.d.	n.d.	3.594±0.008	n.d.	n.d.	n.d.	n.d.	n.d.	n.d.
	R4	n.d.	n.d.	n.d.	n.d.	n.d.	n.d.	5.147±0.016	n.d.	n.d.
	R5	1.521±0.006	n.d.	n.d.	n.d.	n.d.	n.d.	n.d.	n.d.	n.d.
	R6	n.d.	n.d.	n.d.	n.d.	n.d.	n.d.	n.d.	n.d.	n.d.
Black locust	BL1	n.d.	n.d.	n.d.	n.d.	n.d.	n.d.	n.d.	n.d.	n.d.
	BL2	n.d.	n.d.	n.d.	n.d.	n.d.	n.d.	n.d.	n.d.	n.d.
	BL3	n.d.	n.d.	n.d.	n.d.	n.d.	n.d.	n.d.	0.230±0.003	n.d.
	BL4	n.d.	n.d.	n.d.	n.d.	n.d.	n.d.	n.d.	n.d.	n.d.
	BL5	0.708±0.112	n.d.	n.d.	n.d.	n.d.	n.d.	n.d.	n.d.	n.d.
	BL6	n.d.	n.d.	n.d.	n.d.	n.d.	n.d.	n.d.	n.d.	n.d.
Sunflower	S1	0.600±0.004	n.d.	n.d.	n.d.	n.d.	n.d.	n.d.	n.d.	n.d.
	S2	n.d.	0.610±0.006	n.d.	n.d.	n.d.	5.720±0.013	n.d.	n.d.	n.d.
	S3	n.d.	n.d.	n.d.	n.d.	n.d.	n.d.	n.d.	n.d.	n.d.
	S4	n.d.	n.d.	n.d.	1.835±0.008	4.168±0.010	n.d.	n.d.	n.d.	n.d.
	S5	3.376±0.009	n.d.	n.d.	0.632±0.014	2.937±0.010	n.d.	n.d.	n.d.	n.d.
	S6	n.d.	2.308±0.011	n.d.	n.d.	n.d.	n.d.	n.d.	n.d.	0.318±0.07
Buckwheat	B1	n.d.	n.d.	n.d.	n.d.	n.d.	n.d.	n.d.	n.d.	n.d.
	B2	n.d.	n.d.	n.d.	n.d.	n.d.	n.d.	n.d.	n.d.	n.d.
	B3	n.d.	n.d.	n.d.	n.d.	n.d.	n.d.	n.d.	n.d.	n.d.
	B4	n.d.	n.d.	n.d.	n.d.	n.d.	n.d.	n.d.	n.d.	n.d.
	B5	1.179±0.022	n.d.	1.569±0.015	n.d.	n.d.	n.d.	n.d.	n.d.	n.d.
	B6	0.782±0.019	n.d.	0.981±0.011	n.d.	n.d.	n.d.	n.d.	n.d.	n.d.
Mint	M1	n.d.	n.d.	n.d.	n.d.	n.d.	n.d.	n.d.	n.d.	n.d.
	M2	n.d.	n.d.	n.d.	n.d.	n.d.	n.d.	n.d.	n.d.	n.d.
	M3	n.d.	n.d.	n.d.	n.d.	n.d.	n.d.	0.426±0.007	n.d.	n.d.
	M4	n.d.	n.d.	n.d.	n.d.	n.d.	n.d.	n.d.	n.d.	n.d.
	M5	n.d.	n.d.	n.d.	n.d.	n.d.	n.d.	n.d.	n.d.	n.d.
	M6	n.d.	n.d.	n.d.	n.d.	n.d.	n.d.	n.d.	n.d.	n.d.

Table S3. Cont.

Honey Variety		Pesticide residues, ng/g								
		Imidacloprid	Acetamiprid	Lindane	Aldrin	Endrin	Chlordane	Endosulfan	Coumaphos	Chlorpyrifos-methyl
Dandelion	D1	n.d.	n.d.	n.d.	n.d.	n.d.	n.d.	n.d.	n.d.	n.d.
	D2	n.d.	n.d.	n.d.	n.d.	n.d.	n.d.	n.d.	n.d.	n.d.
	D3	n.d.	n.d.	n.d.	n.d.	n.d.	n.d.	n.d.	n.d.	n.d.
	D4	n.d.	n.d.	n.d.	n.d.	n.d.	n.d.	n.d.	n.d.	n.d.
	D5	n.d.	1.385±0.019	n.d.	n.d.	n.d.	n.d.	n.d.	n.d.	n.d.
	D6	0.740±0.002	n.d.	n.d.	n.d.	n.d.	n.d.	n.d.	n.d.	n.d.
Meadow	Md1	n.d.	n.d.	n.d.	n.d.	n.d.	n.d.	n.d.	n.d.	n.d.
	Md2	n.d.	n.d.	n.d.	n.d.	n.d.	n.d.	n.d.	n.d.	n.d.
	Md3	n.d.	n.d.	n.d.	n.d.	n.d.	n.d.	n.d.	n.d.	n.d.
	Md4	n.d.	n.d.	n.d.	n.d.	n.d.	n.d.	n.d.	n.d.	n.d.
	Md5	n.d.	n.d.	n.d.	n.d.	n.d.	n.d.	n.d.	n.d.	n.d.
	Md6	n.d.	n.d.	0.100±0.002	n.d.	n.d.	n.d.	n.d.	n.d.	n.d.
Raspberry	RA1	n.d.	n.d.	n.d.	n.d.	n.d.	n.d.	n.d.	n.d.	n.d.
	RA2	n.d.	n.d.	n.d.	n.d.	n.d.	n.d.	n.d.	n.d.	n.d.
	RA3	n.d.	0.100±0.002	n.d.	n.d.	n.d.	n.d.	n.d.	n.d.	n.d.
	RA4	n.d.	n.d.	n.d.	n.d.	n.d.	n.d.	n.d.	n.d.	n.d.
	RA5	n.d.	n.d.	n.d.	n.d.	n.d.	n.d.	n.d.	n.d.	n.d.
	RA6	n.d.	n.d.	n.d.	n.d.	n.d.	n.d.	n.d.	n.d.	n.d.

Every value is a mean of three determinations (n = 3) ± standard deviations; n.d.-undetectable.

Tables S4. Pearson correlation for physicochemical parameters and minerals of different type of honey

Table S4a. Pearson correlation for physicochemical parameters and minerals of rapeseed honey

Variables	W	S	HMF	ID	L*	a*	b*	C	H	YI	Na	Ca	Mn
W	**1**												
S	0.505	**1**											
HMF	0.469	0.910	**1**										
ID	0.676	0.806	0.924	**1**									
L*	0.001	0.000	0.051	0.096	**1**								
a*	0.040	0.194	0.390	0.384	0.766	**1**							
b*	0.086	0.055	0.165	0.197	0.541	0.563	**1**						
C	0.088	0.060	0.173	0.206	0.553	0.580	1.000	**1**					
H	0.019	0.193	0.376	0.352	0.728	0.983	0.436	0.453	**1**				
YI	0.058	0.061	0.207	0.258	0.842	0.840	0.866	0.876	0.741	**1**			
Na	0.604	0.361	0.544	0.668	0.066	0.180	0.338	0.342	0.116	0.262	**1**		
Ca	0.001	0.349	0.221	0.066	0.340	0.021	0.138	0.135	0.008	0.173	0.002	**1**	
Mn	0.396	0.335	0.530	0.578	0.016	0.112	0.133	0.137	0.079	0.114	0.881	0.064	**1**

W-water content, S- sucrose, ID- diastase activity, HMF- hydroxymethylfurfural, C-chroma, H-hue angle, YI- yellow index, Na-sodium, Ca- calcium, Mn- manganese.

Table S4b. Pearson correlation for physicochemical parameters and minerals of black locust honey

Variables	W	F	S	G/F	G/W	EC	HMF	ID	L*	a*	b*	H	YI	Na	K	Mg	Ca	Mn	Cu	Cd
W	**1**																			
F	-0.046	**1**																		
S	0.789	0.133	**1**																	
G/F	0.450	-0.822	0.094	**1**																
G/W	-0.704	-0.255	-0.814	0.167	**1**															
EC	0.806	0.478	0.802	-0.134	-0.867	**1**														
HMF	0.963	-0.030	0.820	0.344	-0.799	0.791	**1**													
ID	-0.906	0.109	-0.773	-0.316	0.845	-0.739	-0.977	**1**												
L*	0.728	0.613	0.571	-0.184	-0.690	0.926	0.695	-0.617	**1**											
a*	-0.446	0.302	-0.393	-0.122	0.740	-0.401	-0.557	0.715	-0.207	**1**										
b*	0.015	0.294	0.569	-0.314	-0.220	0.171	0.104	-0.037	-0.012	0.235	**1**									
H	0.433	-0.382	0.211	0.244	-0.603	0.307	0.519	-0.675	0.193	-0.960	-0.478	**1**								
YI	-0.405	-0.084	0.147	-0.177	0.200	-0.372	-0.314	0.324	-0.565	0.308	0.831	-0.503	**1**							
Na	0.736	-0.128	0.692	0.165	-0.903	0.718	0.791	-0.875	0.510	-0.908	-0.075	0.834	-0.345	**1**						
K	0.634	-0.095	0.166	0.281	-0.525	0.523	0.618	-0.677	0.586	-0.671	-0.680	0.803	-0.890	0.699	**1**					
Mg	0.342	-0.465	0.148	0.640	0.146	-0.169	0.393	-0.337	-0.116	0.118	0.126	-0.043	0.142	-0.092	-0.003	**1**				

Ca	-0.618	-0.409	-0.585	0.158	0.742	-0.887	-0.536	0.516	-0.794	0.440	0.076	-0.370	0.493	-0.700	-0.558	0.523	**1**			
Mn	0.735	0.596	0.599	-0.194	-0.739	0.948	0.707	-0.642	0.996	-0.274	-0.013	0.249	-0.562	0.570	0.606	-0.164	-0.835	**1**		
Cu	0.330	-0.787	-0.048	0.829	0.124	-0.259	0.360	-0.395	-0.236	-0.258	-0.350	0.406	-0.181	0.151	0.356	0.821	0.420	-0.254	**1**	
Cd	0.584	0.077	0.554	-0.047	-0.847	0.744	0.588	-0.669	0.566	-0.823	-0.182	0.753	-0.456	0.923	0.689	-0.446	-0.867	0.633	-0.156	**1**

W-water Content, G- glucose, F- fructose, S- sucrose, G/F- glucose to fructose ratio, G/W- glucose to water content ratio, EC- electrical conductivity, ID- diastase activity, HMF- hydroxymethylfurfural, L*, a*, b*, C-chroma, H-hue angle, YI- yellow index, Na-sodium, K- potassium, Mg- magnesium, Ca- calcium, Mn- manganese, Cu-copper, Cd-cadmium.

Table S4c. Pearson correlation for physicochemical parameters and minerals of sunflower honey

Variables	G	F	G/F	EC	L*	a*	b*	C	Na	Ca	Mn	Cu	Zn	Pb	Cd
G	**1**														
F	-1.00	**1**													
G/F	0.26	-0.22	**1**												
EC	-0.33	0.32	0.16	**1**											
L*	-0.16	0.14	0.28	0.58	**1**										
a*	-0.36	0.42	-0.02	-0.61	-0.39	**1**									
b*	0.16	-0.13	0.08	-0.90	-0.67	0.71	**1**								
C	0.07	-0.04	0.07	-0.89	-0.65	0.80	0.99	**1**							
Na	-0.13	0.14	-0.86	-0.22	-0.33	0.24	-0.05	0.00	**1**						
Ca	0.36	-0.41	0.16	0.59	0.34	-0.98	-0.61	-0.71	-0.41	**1**					
Mn	0.81	-0.82	0.53	0.85	-0.40	-0.15	0.51	0.82	-0.63	0.31	**1**				
Cu	-0.32	0.29	0.33	0.19	0.82	-0.03	-0.19	-0.16	-0.49	0.05	-0.19	**1**			
Zn	0.83	-0.84	0.30	0.24	0.17	-0.78	-0.38	-0.47	-0.26	0.77	0.84	-0.20	**1**		
Pb	-0.37	0.34	0.28	0.87	0.33	-0.56	-0.62	-0.63	-0.49	0.64	0.82	0.16	0.15	**1**	
Cd	0.87	-0.91	0.03	-0.30	-0.14	-0.55	0.13	0.01	-0.17	0.56	0.86	-0.19	0.77	-0.22	**1**

G- glucose, F- fructose, G/F- glucose to fructose ratio, EC- electrical conductivity, HMF- hydroxymethylfurfural, L*, a*, b*, C-chroma, Na-sodium, Ca- calcium, Mn- manganese, Cu-copper, Zn-zinc, Pb- lead,Cd-cadmium,

Table S4d. Pearson correlation for physicochemical parameters and minerals of buckwheat honey

Variables	W	G	F	S	G/W	EC	ID	L*	a*	b*	C	YI	Mn	Zn	Ni	Cd
W	**1**															
G	0.393	**1**														
F	0.554	0.898	**1**													
S	0.548	0.807	0.935	**1**												
G/W	0.746	0.863	0.874	0.833	**1**											
EC	0.801	0.525	0.596	0.645	0.816	**1**										
ID	0.312	0.443	0.703	0.793	0.427	0.299	**1**									
L*	0.824	0.184	0.261	0.230	0.504	0.743	0.057	**1**								
a*	0.040	0.026	0.045	0.003	0.001	0.021	0.047	0.027	**1**							
b*	0.755	0.535	0.618	0.553	0.775	0.899	0.258	0.785	0.016	**1**						
C	0.755	0.535	0.618	0.553	0.775	0.899	0.258	0.785	0.016	1.000	**1**					
YI	0.526	0.703	0.765	0.685	0.767	0.767	0.406	0.470	0.110	0.893	0.893	**1**				
Mn	0.036	0.036	0.059	0.009	0.002	0.154	0.019	0.624	0.091	0.419	0.169	0.046	**1**			
Zn	0.510	0.219	0.225	0.162	0.428	0.665	0.013	0.819	0.011	0.825	0.825	0.617	0.109	**1**		
Ni	0.081	0.207	0.368	0.520	0.157	0.069	0.822	0.010	0.000	0.019	0.019	0.084	0.046	0.077	**1**	
Cd	0.483	0.829	0.943	0.881	0.796	0.630	0.681	0.273	0.118	0.700	0.700	0.896	0.034	0.315	0.305	**1**

W-Water Content, G- glucose, F- fructose, S- sucrose, G/W- glucose to water content ratio, EC- electrical conductivity, ID- diastase activity, L*, a*, b*, C-chroma, YI- yellow index, Mn-manganese, Zn-zinc, Ni- nickel, Cd-cadmium.

Table S4e. Pearson correlation for physicochemical parameters and minerals of mint honey

Variables	G	F	G/F	HMF	ID	K	Mg	Ca	Mn	Fe	Zn	Ni	Pb	Cd
G	**1**													
F	0.89	**1**												
G/F	0.95	0.89	**1**											
HMF	0.78	0.92	0.73	**1**										
ID	0.65	0.80	0.79	0.64	**1**									
K	0.01	0.07	0.05	0.01	0.18	**1**								
Mg	0.40	0.28	0.24	0.35	0.14	0.30	**1**							
Ca	0.03	0.14	0.01	0.35	0.06	0.04	0.08	**1**						
Mn	0.08	0.01	0.03	0.08	0.01	0.82	0.43	0.11	**1**					
Fe	0.76	0.79	0.80	0.83	0.66	0.00	0.23	0.22	0.16	**1**				
Zn	0.00	0.03	0.02	0.00	0.05	0.86	0.49	0.04	0.70	0.00	**1**			
Ni	0.40	0.26	0.23	0.35	0.11	0.36	0.99	0.09	0.52	0.25	0.53	**1**		
Pb	0.83	0.76	0.76	0.74	0.68	0.01	0.68	0.09	0.17	0.69	0.09	0.65	**1**	
Cd	0.80	0.53	0.73	0.41	0.44	0.02	0.58	0.01	0.17	0.46	0.08	0.56	0.79	**1**

G- glucose, F- fructose, G/F- glucose to fructose ratio, HMF- Hydroxymethylfurfural, ID- diastase activity, K- potassium, Mg- magnesium, Ca- calcium, Mn- manganese, Fe- iron, Zn- zinc, Ni- nickel, Pb- lead, Cd-cadmium.

Table S4f. Pearson correlation for physicochemical parameters and minerals of dandelion honey

Variables	G	F	S	G/F	HMF	ID	L*	a*	b*	Mn	Cu	Fe	Cd
G	**1**												
F	0.625	**1**											
S	0.884	0.512	**1**										
G/F	0.969	0.498	0.904	**1**									
HMF	0.902	0.416	0.938	0.967	**1**								
ID	0.579	0.940	0.572	0.491	0.438	**1**							
L*	0.000	0.250	0.014	0.003	0.012	0.223	**1**						
a*	0.028	0.018	0.031	0.072	0.112	0.025	0.236	**1**					
b*	0.076	0.038	0.101	0.083	0.079	0.035	0.838	0.384	**1**				
Mn	0.476	0.361	0.501	0.391	0.321	0.089	0.237	0.335	0.449	**1**			
Cu	0.180	0.135	0.065	0.197	0.102	0.145	0.080	0.021	0.000	0.801	**1**		
Fe	0.534	0.178	0.804	0.645	0.800	0.267	0.056	0.144	0.070	0.181	0.000	**1**	
Cd	0.055	0.002	0.158	0.111	0.234	0.000	0.034	0.407	0.003	0.433	0.202	0.507	**1**

G- glucose, F- fructose, S-sucrose, G/F- glucose to fructose ratio, HMF- Hydroxymethylfurfural, ID- diastase activity, L*, a*, b*, Mn- manganese, Cu-copper, Fe- iron, Cd-cadmium.

Table S4g. Pearson correlation for physicochemical parameters and minerals of meadow honey

Variables	W	G	F	S	G/F	G/W	EC	L*	a*	b*	H	Na	K	Mg	Fe
W	**1**														
G	0.105	**1**													
F	0.086	0.938	**1**												
S	0.387	0.024	0.000	**1**											
G/F	0.081	0.983	0.957	0.001	**1**										
G/W	0.905	0.358	0.314	0.349	0.312	**1**									
EC	0.968	0.182	0.167	0.252	0.162	0.939	**1**								
L*	0.182	0.824	0.909	0.000	0.879	0.416	0.296	**1**							
a*	0.397	0.483	0.552	0.248	0.431	0.576	0.405	0.569	**1**						
b*	0.571	0.469	0.548	0.016	0.506	0.723	0.712	0.772	0.559	**1**					
H	0.471	0.560	0.630	0.196	0.521	0.676	0.511	0.684	0.975	0.705	**1**				
Na	0.592	0.125	0.134	0.513	0.073	0.610	0.520	0.106	0.591	0.263	0.560	**1**			
K	0.251	0.136	0.078	0.801	0.062	0.320	0.172	0.020	0.400	0.025	0.321	0.694	**1**		
Mg	0.600	0.195	0.269	0.284	0.161	0.642	0.571	0.324	0.852	0.539	0.834	0.796	0.382	**1**	
Fe	0.587	0.029	0.045	0.458	0.009	0.515	0.491	0.044	0.498	0.215	0.459	0.952	0.560	0.799	**1**

W-Water Content, G- glucose, F- fructose, S-sucrose, G/F- glucose to fructose ratio, G/W- glucose to water content ratio, EC- electrical conductivity L*, a*, b*, H-Hue Angle, Na-sodium, K- potassium, Mg- magnesium, Fe- iron.

Table S4h. Pearson correlation for physicochemical parameters and minerals of raspberry honey

Variables	W	S	ID	L*	a*	b*	IPZ	Na	Mn	Cu	Fe	Ni
W	**1**											
S	0.652	**1**										
ID	0.504	0.932	**1**									
L*	0.129	0.067	0.010	**1**								
a*	0.098	0.023	0.044	0.006	**1**							
b*	0.005	0.004	0.006	0.003	0.819	**1**						
IPZ	0.400	0.260	0.079	0.677	0.005	0.143	**1**					
Na	0.000	0.025	0.037	0.270	0.192	0.047	0.061	**1**				
Mn	0.181	0.206	0.089	0.038	0.091	0.015	0.303	0.446	**1**			
Cu	0.079	0.000	0.001	0.368	0.016	0.001	0.119	0.863	0.302	**1**		
Fe	0.222	0.257	0.098	0.645	0.017	0.042	0.812	0.001	0.516	0.005	**1**	
Ni	0.063	0.001	0.002	0.069	0.804	0.748	0.100	0.216	0.019	0.029	0.051	**1**

W-Water Content, S-sucrose, ID- diastase activity, L*, a*, b*, Na-sodium, Mn- manganese, Cu-copper, Fe- iron, Ni- nickel.
